# QM/MM Modeling of the Electronic Structure and Properties of the Fe–S Clusters in *Desulfovibrio desulfuricans* [FeFe]-Hydrogenase

**DOI:** 10.1021/acs.inorgchem.6c01252

**Published:** 2026-06-14

**Authors:** Anna Rovaletti, Meritxell Wu-Lu, Federica Arrigoni, Luca De Gioia, Ulf Ryde, Claudio Greco, Luca Bertini

**Affiliations:** † Department of Earth and Environmental Sciences, University of Milano-Bicocca, Piazza Della Scienza 1, 20126 Milan, Italy; ‡ Department of Chemistry, Technical University of Berlin, 10623 Berlin, Germany; § Department of Biotechnologies and Biosciences, University of Milano-Bicocca, Piazza Della Scienza 2, 20126 Milan, Italy; ∥ Department of Theoretical Chemistry, Chemical Centre, Lund University, P.O. Box 124, SE-221 00 Lund, Sweden

## Abstract

[FeFe]-hydrogenases are highly efficient enzymes in the reversible catalysis of molecular hydrogen production and oxidation. Their active site, the H-cluster, consists of a [4Fe–4S]_H_ subcluster linked to a binuclear [2Fe]_H_ organometallic unit. Many [FeFe]-hydrogenases, such as the one from *Desulfovibrio desulfuricans* (DdHydAB), possess accessory Fe–S clusters (F and F′) that mediate electron transfer. This study employs hybrid quantum mechanics/molecular mechanics (QM/MM) methods to characterize the electronic structure and thermodynamic landscape associated with redox and protonation events in the complete Fe–S cluster network of DdHydAB. Our calculations indicate that the F′ cluster plays a key role in the initial reduction of the oxidized resting state, acting as the preferential site for the accumulation of the first electron. Analysis of protonated states, upon reduction events, reveals a strong correlation between protonation and electron transfer (PCET), with protonation at the H-cluster inducing electron transfer from the F′ cluster to the H-cluster. Calculations indicate that the formation of a terminal hydride is energetically favored over ADT protonation, and subsequent isomerization to a bridging hydride (μ-H) is further stabilizing, albeit potentially kinetically limiting. The study highlights how accessory clusters influence the electronic distribution and redox properties of the H-cluster, underscoring the importance of considering the entire Fe–S cluster system for a complete understanding of the catalytic mechanism of [FeFe]-hydrogenases.

## Graphical abstract

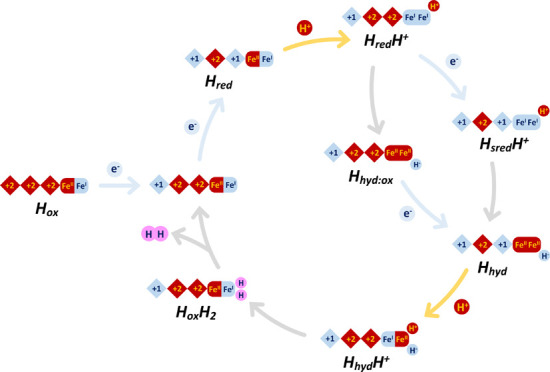

## Introduction

In nature, molecular hydrogen serves as a central metabolic component for various bacterial and archaeal microorganisms,^1,2^ and recent studies suggest that it was likely fundamental to the origin of primordial microbial life.^3^

Three classes of metalloenzymes phylogenetically unrelated, [FeFe]-hydrogenases and [NiFe]-hydrogenases and [Fe]-hydrogenases, are crucial in this type of metabolism and are capable of expediently shifting the equilibrium

$$H_{2}⇄\left[H^{+}+H^{-}\right]⇄{2H}^{+}+{2e}^{-}$$

by modulating the acidity of molecular hydrogen and thus catalyzing the oxidation of H_2_ or the reduction of protons.^4^ Their catalytic efficiency has made hydrogenases the subject of extensive investigation, particularly in the context of hydrogen-based energy technologies.^5^ Among them, [FeFe]-hydrogenases are generally catalytically biased toward fermentative H_2_ generation and are predominantly found in obligate anaerobic bacteria, some phototrophic eukaryotes^6,7^ and recently in archaea.^8^ Their catalytic activity originates from a highly conserved active site, the H-cluster, a unique [6Fe–6S] cofactor consisting of a canonical [4Fe–4S] cubane linked via a cysteine residue to a binuclear organometallic [2Fe]_H_ subsite. The latter contains two iron atoms, referred to as proximal (Fe_P_) and distal (Fe_D_) with respect to the [4Fe–4S]_H_ cubane, coordinated by CO and CN^–^ ligands and bridged by an azadithiolate (ADT) group, forming a [Fe_2_(CN)_2_(CO)_3_(ADT)]^2–^ unit.^9^ This unusual coordination environment supports exceptionally high turnover frequencies, also due to the peculiar inverted square-pyramidal, edge-sharing geometry of the diiron center (the so-called “rotated state”), which leaves an open coordination site at Fe_D_, available for substrate binding.^10^

While the H-cluster itself is structurally conserved, the diversity of [FeFe]-hydrogenases^6,8,11^ arises from the presence of accessory Fe–S clusters that enable electron transfer processes to and from the outside of the enzyme. The F-domain is the portion of the enzyme that can hold auxiliary Fe–S clusters, whose number depends on the type of microorganism considered.^12–16^

In sulfate-reducing bacteria of the *Desulfovibrio* genus, hydrogen metabolism is central to cellular energy conservation.^17–19^ [FeFe]-hydrogenase from *Desulfovibrio desulfuricans* (DdHydAB) is one of the first structurally characterized members of this enzyme family.^20^ According to Meyer classification^13^ DdHydAB is an M2 enzyme containing two accessory [4Fe–4S] clusters, F and F′ where F′ is the more external cluster located near the enzyme’s surface and F is situated internally between F′ and the H-cluster. These Fe–S clusters are arranged in an electron-transfer chain connecting the H-cluster to the protein surface through edge-to-edge distances compatible with rapid electron transfer (Figure 1).

**Figure 1:**
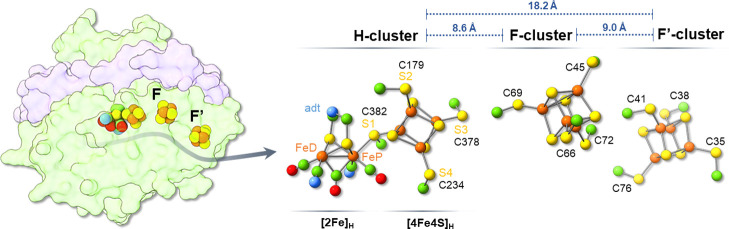
Left: structure of *Desulfovibrio desulfuricans* [FeFe]-hydrogenases (PDB ID 1HFE), with the large and small subunits colored in green and purple, respectively. The H-cluster and accessory [4Fe–4S] clusters (F, F′) are shown as spheres. Right: ball and stick representation of the QM portion of our QM/MM system, comprising all the Fe–S clusters. Color scheme: orange, Fe; yellow, S; blue, N; red, O; green, C.

Extensive spectroscopic and computational studies have established that catalysis proceeds through a sequence of redox and protonation states of the H-cluster^16,21–24^

**H**_**ox**_ represents the starting point and the most oxidized form of the H-cluster, characterized by a mixed-valent Fe­(I)­Fe­(II) state of the [2Fe]_H_ dinuclear cluster and a [4Fe–4S]_H_ in the diamagnetic oxidized state (2Fe­(II)­2Fe­(III)). **H**_**ox**_ can be reduced to the **H**_**red**_ state with a reduced [4Fe–4S]_H_ cubane, which can be protonated at ADT to **H**_**red**_ **H** ^**+**^ state resulting in a Fe­(I)­Fe­(I) [2Fe]_H_ state with an oxidized [4Fe–4S]_H_ cluster.^25^ A p*K*_a_ of 7.2 has been measured for the **H**_**red**_ **/H**_**red**_ **H** ^**+**^ couple for [FeFe] hydrogenase from *Chlamydomonas reinhardtii* CrHydA1 and at pH < p*K*_a_, **H**_**ox**_ reduction and protonation are coupled.^25^ In this case the presence of a proton on the [4Fe–4S]_H_ cubane has been proposed at the **H**_**red**_ level,^26,27^ with this “regulatory” proton suggested to remain associated with the cluster during catalytic turnover. In very recent work, the persistence of protonated spectroscopic signatures (previously attributed to the regulatory proton-transfer pathway) even upon its disruption has led to the proposal that these features instead arise from a Zundel-type ion (H_5_O_2_^+^) formed between two conserved water molecules along the catalytic proton-transfer pathway.^28^

**H**_**red**_ **H** ^**+**^ can be further reduced and reversibly converted to a hydride-containing **H**_**hyd**_ state, characterized by a terminal hydride and an oxidized Fe­(II)­Fe­(II) [2Fe]_H_ cluster.^29–31^

Notably, the assignment of the **H**_**hyd**_ state remains subject to debate, as EPR and IR studies have revealed heterogeneity in the observed spectral signatures and the possible presence of multiple substates. Nevertheless, the available evidence consistently supports a description involving a 2Fe­(II)–H^–^ unit coupled to a reduced [4Fe–4S]_H_ cluster.^32^ In this context, spectroelectrochemical IR microscopy studies on [FeFe]-hydrogenase crystals^33^ have demonstrated the existence of a **H**_**hyd**_ state family.

The transition between **H**_**red**_ **H** ^**+**^ and **H**_**hyd**_ involves two sequential steps, one reduction and one protonation which depend on the redox potential and pH.^31^ Spectroscopic studies have shown that, under illumination, both **H**_**red**_ **H** ^**+**^ and the one-electron reduced counterpart **H**_**sred**_ **H** ^**+**^ in CrHydA1 from *C. reinhardtii* (which lacks the F-clusters) can reversibly convert to a hydride-containing isomer distinct from the previously characterized **H**_**hyd**_ state.^30,31,34^ In one case, the system first adopts a **H**_**sred**_ **H** ^**+**^ reduced form of **H**_**red**_ **H** ^**+**^, with the [4Fe–4S]_H_ cluster reduced and the ADT amine protonated. This intermediate then undergoes a rearrangement in which the proton is transferred from ADT to Fe_D_ forming a terminal hydride. In an alternative pathway, the proton transfer to Fe_D_ occurs first, forming a hydride intermediate called **H**_**hyd/ox**_, followed by electron transfer to yield the fully reduced **H**_**hyd**_ state also called **H**_**hyd/red**_. At this point two electrons and one proton are accumulated at the H-cluster and the subsequent protonation of the ADT amine generates a proton–hydride pair in the **H**_**hyd**_ **H** ^**+**^ state,^35^ which can rapidly recombine to **H**_**ox**_ **H**_**2**_, which then can release molecular hydrogen.

Besides active site chemistry, the role and redox features of the F-clusters in different hydrogenases have also been experimentally investigated, and their possible implications in catalysis, catalytic bias, and oxygen resistance have been discussed.^12,18,36–39^ ^,^ In this regard, most density functional theory (DFT) studies have extensively focused on the H-cluster, while the role of the accessory Fe–S clusters has remained largely unexplored from a computational perspective.^30,40–47^

A more complete description requires a quantum mechanical and molecular mechanics (QM/MM) treatment in which the entire Fe–S network is explicitly included in the region described at DFT level. This strategy was previously applied to DdHydAB, including the H-cluster together with the F and F′ accessory clusters, and revealed significant long-range electronic communication within the three-cluster ensemble, with important implications for H_2_ binding and activation in oxidized states.^51^ More in detail, along with the unambiguous assignment of a mixed Fe­(I)­Fe­(II) state to the catalytic core of **H**_**ox**_, it was revealed that **H**_**ox**_ is capable of efficiently binding H_2_, thereby initiating the process of H_2_ oxidation catalysis.

A significant finding was that H_2_ binding promotes electron transfer from the H-cluster to the solvent-exposed F′-cluster, suggesting a functional role of long-range electronic coupling in H_2_ activation.^44,51^ Then, cyanide ligands were also shown to be crucial for H-cluster redox tuning, enabling access to the Fe­(I)­Fe­(II) state upon one-electron oxidation.^49,52^ Building on these findings, the present work employs a QM/MM framework to investigate a series of reduced and protonated states of DdHydAB. The corresponding electronic structures and energetics will be discussed in light of recent advances in the structural and electronic characterization of the known intermediates. The rationale behind this study is 2-fold. On one hand, a detailed understanding of the electronic structures of several intermediates can be achieved with the help of computational approaches. On the other hand, in metalloenzymes containing multiple clusters,^53^ the effect of seemingly nonessential parts of the catalytic site is often simplified or overlooked in computational analysis aiming at defining catalytic mechanisms and the associated energetics. However, in these systems, where the energy differences between competing states are often small, even distal metal centers can modulate the redox landscape and relative stability of intermediates, thereby influencing mechanistic interpretations.

## Results and Discussion

Before presenting the results, we briefly clarify the nomenclature adopted in this work. The conventional naming of H-cluster states, established in the early 2000s, is based on the redox states of the [2Fe]_H_ subcluster and the [4Fe–4S]_H_ cubane.^22,54^

However, this convention does not explicitly include the accessory Fe–S clusters. Since elucidating the role of the F′ and F clusters is a key objective of this study, we extend the conventional nomenclature by explicitly specifying their redox states. The list of all states with their full name and assignments is reported in Table S1 in the Supporting Information.

### Stepwise Reduction of DdHydAB

The oxidized resting state of the H-cluster, **H**_**ox**_, has been characterized extensively.^55–58^ Electron paramagnetic resonance (EPR) studies assign the rhombic signal to an H-cluster in which [4Fe–4S]_H_ is oxidized and the [2Fe]_H_ subcluster is in the paramagnetic mixed-valent Fe­(II)­Fe­(I) state.^25,59^ This redox configuration is proposed to be conserved across different enzymes, including DdHydAB, CpHydA1 from *Clostridium pasteurianum* (which contains, beyond the H-cluster, three accessory [4Fe–4S] clusters and one [2Fe–2S] cluster) and CrHydA1 from *C. reinhardtii* (which is devoid of any accessory Fe–S clusters).^36,37^ Our investigation starts from the fully oxidized state of DdHydAB, denoted **F′**_**ox**_ **F**_**ox**_ **H**_**ox**_. This species is well reproduced by our computational model, being characterized by an H-cluster with a mixed-valence binuclear core (Fe_P_–Fe_D_ distance of 2.57 Å compared with 2.57 and 2.55 Å in 1HFE dimer) and by all the [4Fe–4S] clusters in the 2Fe­(II)­2Fe­(III) oxidation state (see Table S1).

The one-electron reduction of **F′**_**ox**_ **F**_**ox**_ **H**_**ox**_ yields a **F′**_**red**_ **F**_**ox**_ **H**_**ox**_ species, in which the electronic structure of the H-cluster, including both the [4Fe–4S]_H_ cubane and the [2Fe]_H_ subsite, remains unchanged (see Figure 2). The differences in geometries, charges and spin populations of the H-cluster in **F′**_**ox**_ **F**_**ox**_ **H**_**ox**_ and in the one-electron reduced counterpart **F′**_**red**_ **F**_**ox**_ **H**_**ox**_ are negligible (maximum deviations are 0.05 Å for the Fe_P_–Fe_D_ distance, 0.01 and 0.03 for the charge and spin population of Fe_D_, see Table S1). This result is in nice agreement with spectro-electrochemical redox titration results reported by Rodriguez-Maciá et al.^36^ showing that, for DdHydAB in which ADT is substituted with PDT (propane dithiolate), the F′-cluster has the highest midpoint potential (−380 mV) among all the Fe–S clusters of DdHydAB­(PDT), being the thermodynamically preferred site for the first one-electron reduction of the protein, in line with previous theoretical studies on the same system using a comparable QM/MM framework.^60^ A second one-electron reduction leads to electron localization at the H-cluster, yielding a species consistent with the state reported in the literature as **F′**_**red**_ **F**_**ox**_ **H**_**red**_, which is EPR-silent due to strong exchange coupling (Figure 2).^22^ In principle, such a reduction can take place either in the cubane subsite, leading to the [4Fe–4S]^+^_H_–Fe­(I)­Fe­(II) configuration, or in the binuclear subsite, yielding the [4Fe–4S]^+2^_H_–Fe­(I)­Fe­(I) analogue. Our QM/MM results indicate that **F′**_**red**_ **F**_**ox**_ **H**_**red**_ features the [2Fe]_H_ subcluster in the Fe­(I)­Fe­(II) state, with a Fe_P_–Fe_D_ distance of 2.57 Å. To further support this assignment, a calculation was initiated from an electronic configuration featuring a reduced F cluster and an oxidized [4Fe–4S]_H_ subcluster; however, it converged to a state with an oxidized F cluster and a reduced [4Fe–4S]_H_ subcluster, indicating a preference for this redox distribution (see Figure S5). Experimental data for this enzyme (DdHydAB­(PDT)) show that these two electronic structures exist in redox equilibrium, as reflected by their very similar reduction potentials: −380 mV for the **F′**_**red**_ **F**_**ox**_ **H**_**ox**_ **/F′**_**red**_ **F**_**red**_ **H**_**ox**_ couple and −405 mV for the **F′**_**red**_ **F**_**ox**_ **H**_**ox**_/**F′**_**red**_ **F**_**ox**_ **H**_**red**_ couple.^36^ The difference of only 25 mV corresponds to a free energy difference of less than 1 kcal/mol, indicating that the two redox distributions are nearly isoenergetic.

**Figure 2:**
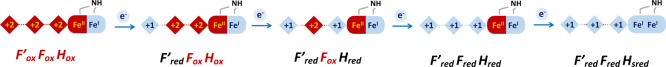
Schematic representation of the three [4Fe–4S] clusters (F′, F, and [4Fe–4S]_H_) and the [2Fe]_H_ subcluster in the investigated redox states. Cluster oxidation states are color-coded (red = oxidized, blue = reduced). The sequence starts from the fully oxidized state (F′_ox_F_ox_H_ox_, with the [2Fe]_H_ unit in the Fe­(I)­Fe­(II) configuration) and proceeds through successive reductions: **F′**_**red**_ **F**_**ox**_ **H**_**ox**_, **F′**_**red**_ **F**_**ox**_ **H**_**red**_, **F′**_**red**_ **F**_**red**_ **H**_**red**_, and finally **F′**_**red**_ **F**_**red**_ **H**_**sred**_.^61,62^

A further one-electron reduction of **F′**_**red**_ **F**_**ox**_ **H**_**red**_ gives rise to a reduced species **F′**_**red**_ **F**_**red**_ **H**_**red**_ that is characterized by a mixed-valence bimetallic center and by three reduced cubanes (Figure 2). For the DdHydAB­(PDT) system, reduction to **F′**_**red**_ **F**_**red**_ **H**_**red**_ has been reported to occur at −495 and −520 mV when starting from **F′**_**red**_ **F**_**red**_ **H**_**ox**_ or **F′**_**red**_ **F**_**ox**_ **H**_**red**_, respectively.^36^ A fourth reduced species has also been characterized, corresponding to the super-reduced form **F′**_**red**_ **F**_**red**_ **H**_**sred**_ (Figure 2). This species is distinguished by a fully reduced binuclear Fe­(I)­Fe­(I) subsite and a reduced cubane.^61,62^

### Protonation and PCET Events

It is reasonable to assume that protonation of the active site is triggered by (or concomitant with) its one-electron reduction. Thus, we considered the protonation properties of the one-electron reduced **F′**_**red**_ **F**_**ox**_ **H**_**ox**_ state (Figure 3, top), by taking into account the following putative proton binding sites of the H-cluster: (i) the four sulfur atoms of the Cys residues bound to [4Fe–4S]_H_, labeled S1, S2, S3 and S4 (Figure 3, bottom), and corresponding to Cys382, Cys179, Cys378 and Cys234 (numbering referred to PDB 1HFE); (ii) the N atom of ADT; (iii) Fe_D_ in terminal position (t-H), both axial (t-H­(a)) and equatorial (t-H­(e)), and (iv) Fe_P_–Fe_D_ bridging position (μ-H).

**Figure 3:**
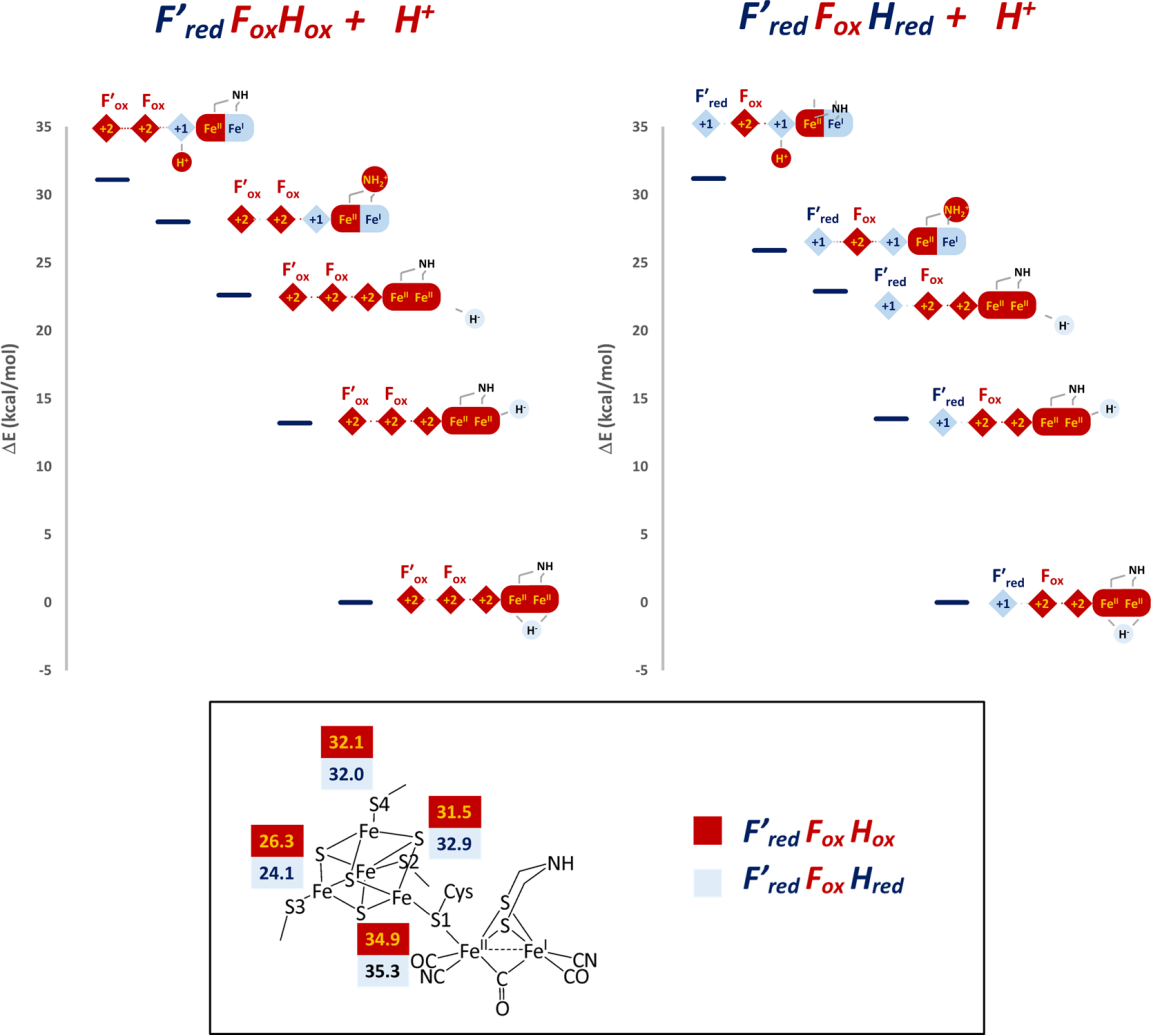
Top: Energetic preference for **F′**_**red**_ **F**_**ox**_ **H**_**ox**_ + H^+^ and **F′**_**red**_ **F**_**ox**_ **H**_**red**_ + H^+^ considering their various forms (protonation of [4Fe–4S]_H_, 2­[Fe]_H_ diiron core or ADT). Energy differences are given in kcal/mol. Bottom: Relative energies (in kcal/mol) are shown for the four protonated forms at [4Fe–4S]_H_ for the two states.

Protonation of the [4Fe4S]_H_ subcluster has been included since spectroscopic investigations of the protonation and reduction dynamics at the H-cluster led to the proposal of the presence of two independent proton pathways in [FeFe]-hydrogenases: the catalytic proton channel, which transports protons to the diiron site via ADT, and a mechanism that promotes protonation at the [4Fe–4S]_H_ cluster through a regulatory proton transfer pathway.^63^ From Figure 3 we can readily observe that:

- The most stable protonated form features a μ-H, corresponding to an isomer in which the originally bridging CO ligand (μ-CO) becomes terminal and adopts an apical coordination.
- The protonation of the initial **F′**_**red**_ **F**_**ox**_ **H**_**ox**_ state at S1–S4 sites of [4Fe–4S]_H_ triggers an electron transfer from the F′ cluster to the H-cluster, suggesting here a genuine proton-coupled electron transfer (PCET) step.
- The structure with protonated ADT is 25.9 kcal/mol less stable than the μ-H state. Also in this state an electron is transferred from the F′ cluster to [4Fe–4S]_H_.
- Protonation of the [2Fe]_H_ cluster diiron core (to form either a terminal or bridging hydride), is also accompanied by a F′-to-active site electron transfer and by the oxidation of both the [4Fe–4S]_H_ and binuclear cluster, which becomes Fe­(II)­Fe­(II).
- Both t-H­(a) and t-H­(e) retain a hydride character. t-H­(a) corresponds to a catalytic intermediate, in which the hydride is oriented toward the ADT nitrogen. In the t-H­(e) form, the hydride occupies a position originally associated with a CO ligand, implying a structural rearrangement of the binuclear cluster and resulting in a species that is less stable by 9.4 kcal/mol with respect to t-H­(a).

The observation that protonation events directly imply electron movements is indicative of a strong correlation between protonation and redox events (i.e., PCET), which has been widely recognized as a fundamental strategy forming the basis of [FeFe]-hydrogenases thermodynamic success, although difficult to be experimentally resolved.^22^

In detail, when protonation of **F′**_**red**_ **F**_**ox**_ **H**_**ox**_ occurs either at ADT or at S1–S4 sites, one electron is transferred from F′ to [4Fe–4S]_H_ giving an **F′**_**ox**_ **F**_**ox**_ **H**_**red**_ **H** ^**+**^ state. These species are all characterized by a mixed-valence Fe­(I)­Fe­(II) state and a reduced [4Fe–4S]_H_ cubane. Proton binding to one (or both) metal center(s), instead, induces an electron transfer toward the protonated [2Fe]_H_ forming **F′**_**ox**_ **F**_**ox**_ **H**_**hyd/ox**_ species with an oxidized [4Fe–4S]_H_ cubane (see Table S1 for further details).

Protonation of ADT in the **F′**_**red**_ **F**_**ox**_ **H**_**ox**_ state turned out to be energetically preferred over protonation of the one of S atoms, even though protonation of S3 is nearly isoenergetic (energy difference of 0.4 kcal/mol in favor of ADT, see Figure 3). Protonations of S4 and S2 are 5.8 and 8.6 kcal/mol less favored than that of S3, respectively. Finally, protonation at S1 is energetically disfavored by 5.2 kcal/mol compared to S3. These results are in good agreement with a recent study that identified S3 as the best candidate for protonation at [4Fe4S]_H_, by comparing experimental and DFT-calculated IR spectra of HydA1 and CpI [FeFe]-hydrogenases in their apo-forms featuring a reduced H-cluster.^63^

Overall, our QM/MM results show that protonation events at the [4Fe4S]_H_ subsite could be, in principle, thermodynamically accessible, since they are associated with energy comparable to ADT protonation (Figure 3 and Table S1).

For what concerns protonation of the reduced state **F′**_**red**_ **F**_**ox**_ **H**_**red**_, the energy ranking among the various forms exactly resembles that observed for **F′**_**red**_ **F**_**ox**_ **H**_**ox**_: protonation at the [4Fe–4S]_H_ subcluster and at ADT are higher in energy than protonation at the [2Fe]_H_ diiron core, with the μ-H isomer being the most stable. In all cases, F′ is found to be reduced. Notably, also in this case, a subsequent protonation of **H**_**red**_ **H** ^**+**^ is able to trigger electron transfer from the reduced F′ cluster to the H-cluster, yielding a species of the **F′**_**ox**_ **F**_**ox**_ **H**_**hyd**_ **H** ^**+**^ type. This observation is consistent with the involvement of PCET processes in the enzymatic function.

### On the Electronic Structure of the **H**_**red**_ **H** ^**+**^ State

For **F′**_**red**_ **F**_**ox**_ **H**_**red**_ **H** ^**+**^ electronic structure obtained upon ADT protonation, we observed a deviation from the electronic configuration previously proposed on the basis of both experimental and computational studies.^36,45^ Indeed, in our QM/MM scheme ADT protonation of **H**_**red**_ does not induce the expected [4Fe–4S]_H_-to-[2Fe]_H_ electron transfer within the H-cluster that would yield [4Fe–4S]_H,ox_[Fe­(I)­Fe­(I)] state expected for **H**_**red**_ **H** ^**+**^. Instead, calculations consistently converged to a species in which the [2Fe]_H_ subsite remains in the Fe­(I)­Fe­(II) oxidation state. Notably, in a recent DFT study employing a cluster approach that explicitly included the H-cluster, elements of the second coordination sphere, and key residues along the catalytic proton transfer pathway, we were able to reproduce this intracluster electron transfer when using hybrid functionals.^45^

To probe this discrepancy within our current QM/MM scheme, we carried out additional calculations to evaluate whether an Fe­(I)­Fe­(I) configuration of the **H**_**red**_ **H** ^**+**^ state might represent a previously unexplored electronic minimum. The analysis was performed for the **F′**_**red**_ **F**_**ox**_ **H**_**red**_ **H** ^**+**^ species.

A summary of the tests performed is given below, while full computational details are reported in the Supporting Information. We examined:

- The possible dependence on the density functional by repeating the QM/MM calculations with B3LYP, PBE0, TPSSh, and M06.
- The influence of the initial electronic guess by starting geometry optimizations from both more oxidized and more reduced reference structures.
- The possibility of accessing the alternative Fe­(I)­Fe­(I) configuration through excited-state explorations.
- The effect of enlarging the QM region to include nearby residues potentially stabilizing the protonated ADT.

In all cases, the QM/MM calculations consistently converged to the same electronic description, namely a reduced [4Fe–4S]_H_ cubane coupled to a Fe­(I)­Fe­(II) diiron center. The alternative [4Fe–4S]_H,ox_[Fe­(I)­Fe­(I)] state configuration previously suggested could not be stabilized within this framework.

To further assess whether this behavior might originate from the specific protein configuration underlying the QM/MM setup (originally constructed and equilibrated for the **F′**_**ox**_ **F**_**ox**_ **H**_**ox**_ state) we removed the protein environment and performed DFT cluster calculations on the sole Fe–S clusters. Two scenarios were considered: a minimal “bare” cluster model and an extended model including nearby residues, each treated both in vacuum and with dielectric embedding (COSMO). In this reduced representation, both electronic configurations could be obtained, with their relative stabilization strongly dependent on the model definition and the treatment of environmental effects.

Remarkably, noticeable variations in the HOMO–LUMO gaps were observed, indicating a delicate balance between closely lying electronic states (see Figure S4). Altogether, these findings suggest that, for **H**_**red**_ **H** ^**+**^, the [4Fe–4S]_H,ox_[Fe­(I)­Fe­(I)] and [4Fe–4S]_H,red_[Fe­(I)­Fe­(II)] descriptions correspond to distinct but energetically proximate minima, whose relative stability is highly sensitive to the electrostatic environment and methodological details.

In principle, **H**_**red**_ **H** ^**+**^ lies along the pathway between the fully oxidized and fully reduced states. From a computational perspective, this intermediate reduction level makes it inherently more delicate to describe, as alternative electronic configurations may become energetically competitive. In contrast, the fully oxidized and fully reduced species display more well-defined electronic structures and tend to be reproduced more consistently across different computational models and levels of theory.

### Hydride Characterization

As previously discussed, addition of one or two electrons to DdHydAB does not alter the energetic ranking of the protonated states of the H-cluster. In particular, hydride species assignable to **H**_**hyd/ox**_ are consistently formed in both cases, with terminal hydrides being significantly less stable than their bridging counterparts, irrespective of the reduction level (Figure 3). On this basis, a similar ordering may be reasonably expected to hold also for the protonation of the further reduced **H**_**sred**_ state. Accordingly, we did not perform a full speciation analysis for this species, and instead focused on protonated structures plausibly involved in efficient catalytic turnover (see next paragraph). Given the consistent energetic trends observed, the properties and relative energetics of these hydride species will be discussed in a unified manner.

In μ-H forms the hydride is equally shared between the two metal centers (Fe_D_–H and Fe_P_–H distances in the range of 1.65–1.70 Å). This species is predicted to be extremely low in energy, being ∼13 kcal/mol more stable than t-H­(a). This value is in line with those previously calculated by Finkelmann et al.^64^ and Bruschi and co-workers.^65^ A direct consequence of the high stability of μ-H is that its formation would cause an increase in the overall catalytic energetic span^66^ possibly lowering turnover frequency. This aspect has no direct experimental evidence but studies on [FeFe]-hydrogenases model complexes indicate that the μ-hydride state is often very difficult to reduce.^67–69^

Indeed, as previously noted, a fast H_2_ turnover is compatible with the conservation of a μ-CO along the whole catalysis. Reiher and co-workers^40^ calculated a direct t-H­(a) → μ-H isomerization pathway entailing a large energy barrier (in the range of 25–29 kcal/mol depending on the model size), which avoids the disruption of the salt bridge between terminal CN^–^ ligand and the side chain of the Lys237 and, thus, the intermediate formation of an equatorial hydride, t-H­(e) (see Figure S3). Alternative isomerization processes, occurring via sequential Bailar or Ray–Dutt rotation(s), have been proposed for simple Fe_2_S_2_ systems,^70^ although they neglect the effect exerted by the protein matrix on H-cluster flexibility. According to these pathways, t-H­(e) should instead form as an intermediate structure, by temporarily breaking the CN^–^ interaction with the protein. In light of these considerations, such an t-H­(e) intermediate is unlikely populated in the enzyme, since its formation would entail (in principle) a high-energy process. Indeed, according to our calculations, t-H­(e) is ∼9.5 kcal/mol less stable than t-H­(a). Overall, our results, together with those reported in the literature, support that the isomerization of t-H­(a) to μ-H should not be a facile step, irrespective of the mechanism invoked.

### H_2_ Evolution

An alternative pathway to proton relay at the **F′**_**red**_ **F**_**ox**_ **H**_**red**_ **H** ^+^ stage, leading to formation of the above-described **H**_**hyd/ox**_ hydride species, is represented by a subsequent reduction of DdHydAB to yield the **F′**_**red**_ **F**_**ox**_ **H**_**sred**_ **H** ^+^ state. This species is characterized by a protonated ADT ligand and an H-cluster in which both the [4Fe–4S]_H_ cubane and the [2Fe]_H_ subsite are reduced, the latter adopting a Fe­(I)­Fe­(I) electronic configuration. In this species, the Fe_P_–Fe_D_ bond is 2.61 Å. Moving the proton from ADT to Fe_D_ leads to the formation of a species that can be assigned to **F′**_**red**_ **F**_**ox**_ **H**_**hyd**_, featuring a terminal hydride with the diiron core formally oxidized to the Fe­(II)­Fe­(II) state and a reduced cubane. Due to the oxidation of [2Fe]_H_, the distance between Fe_P_–Fe_D_ shrinks to 2.55 Å and turns out to be 19.0 kcal/mol more stable than **F′**_**red**_ **F**_**ox**_ **H**_**sred**_ **H** ^**+**^ (Figure 4). Once the **F′**_**red**_ **F**_**ox**_ **H**_**hyd**_ species is formed, another protonation at the ADT position (**F′**_**red**_ **F**_**ox**_ **H**_**hyd**_ **H** ^**+**^) leads to a rearrangement in the electronic distribution ([4Fe–4S]_H_ reduced and [2Fe]_H_ in the Fe­(I)­Fe­(II) state with an apical hydride ion at Fe_D_). Here we also converged a higher-energy solution (+7 kcal/mol) featuring a Fe­(II)­Fe­(II) [2Fe]_H_ subcluster and a reduced cubane (see Figure S5 for details). Again, proton transfer from ADT to the apical hydride ion at the Fe_D_ is thermodynamically favored, with an energy gain of 19.2 kcal/mol. This leads to the formation of a H_2_ molecule (**F′**_**red**_ **F**_**ox**_ **H**_**ox**_ **H**_**2,**_ H–H atomic distance of 0.78 Å), which is found to be weakly coordinated to the Fe_D_ atom with a binding energy of −0.4 kcal/mol with Fe_D_–H distances of 1.68 Å and 1.74 Å, respectively. According to the spin populations the redox state of [2Fe]_H_ is Fe^+1.5^Fe^+1.5^ (atomic charges and spin populations on the two Fe ions are very similar, see Table S1). This assignment is consistent with previous computational results on both **F′**_**red**_ **F**_**ox**_ **H**_**ox**_ **H**_**2**_ models and biomimetic analogues.^68^ It should be remembered that neither **F′**_**red**_ **F**_**ox**_ **H**_**ox**_ **H**_**2**_ nor **F′**_**red**_ **F**_**ox**_ **H**_**hyd**_ **H** ^**+**^ have ever been experimentally observed, and their electronic structure can therefore only be inferred from quantum chemical calculations. Dissociation of H_2_ then leads back to **F′**_**red**_ **F**_**ox**_ **H**_**ox**_.

**Figure 4:**
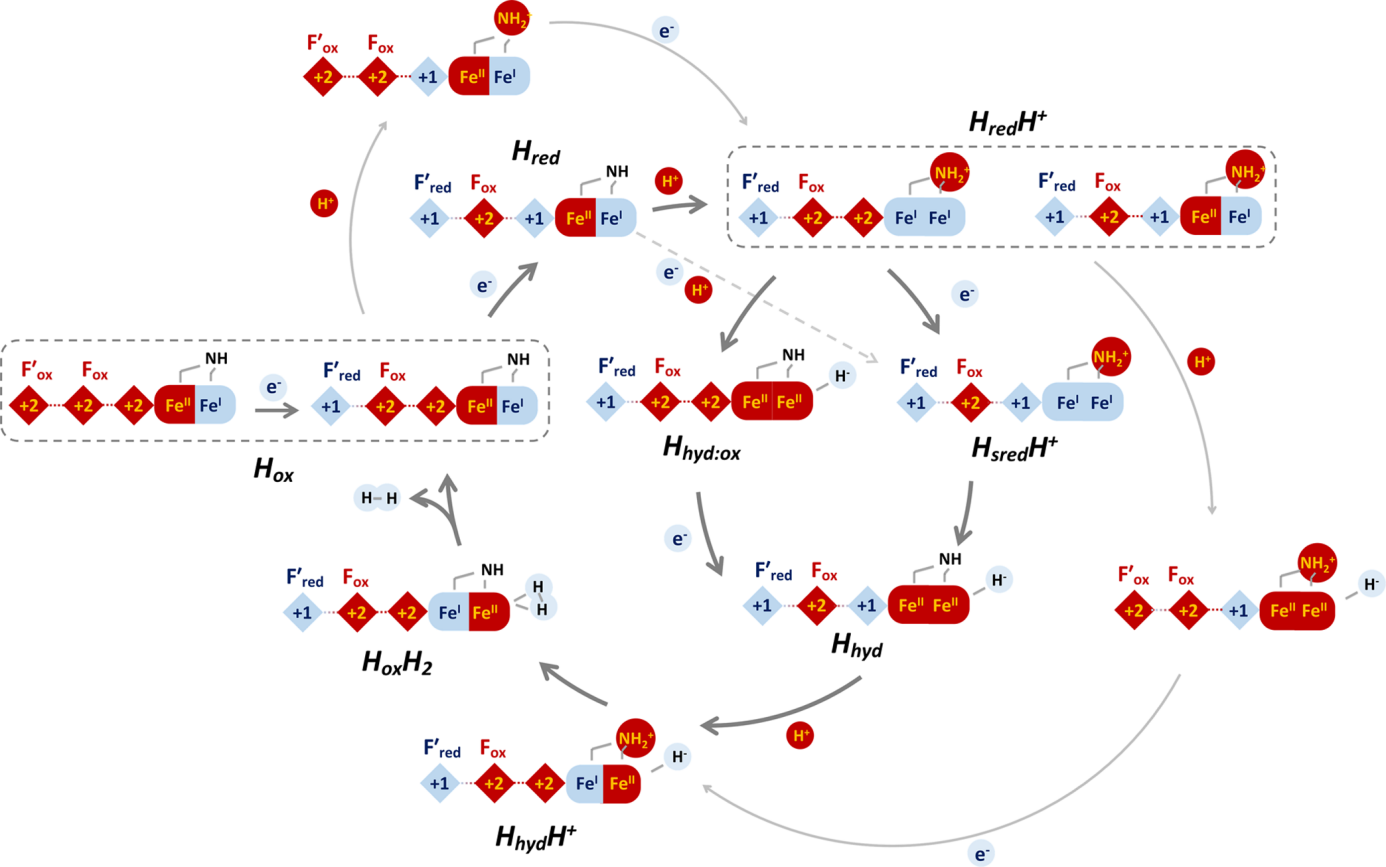
Summary of the H_2_ production or reduction catalytic cycle of DdHydAB based on QM/MM computations. The dashed line connecting H_red_ and H_sred_H^+^ can be interpreted as representing the concerted proton–reduction step described in ref 24.

### Thermodynamic Overview on Redox and Protonation Events

The thermodynamic picture emerging from our QM/MM analysis allows us to delineate the redox and protonation undergone by DdHydAB (Figure 4). More in detail, a first incoming electron preferentially localizes on the F′ cluster, yielding **F′**_**red**_ **F**_**ox**_ **H**_**ox**_. This assignment is consistent with experimental EPR observations on the DdHydAB­(PDT) variant, in which the partially reduced state was attributed to **F′**_**red**_ **F**_**ox**_ **H**_**ox**_ based on the absence of spin coupling between the reduced F cluster and the paramagnetic **H**_**ox**_ **(PDT)** state of the H-cluster.

Upon addition of a second electron, our calculations indicate preferential reduction of the [4Fe–4S]_H_, leading to **F′**_**red**_ **F**_**ox**_ **H**_**red**_. Although previous studies suggested that reduction of the proximal F cluster and of the H-cluster cubane might be nearly isoenergetic, our results favor localization at the H-cluster. This preference can be qualitatively rationalized in terms of electrostatic effects and minimization of Coulombic repulsion among clusters. The three Fe–S clusters are approximately aligned and equidistant; when oxidized, each carries a formal charge of −2, becoming −3 upon reduction. Being the F′–F and F–H-cluster (∼9 Å) separation roughly half than F′–H-cluster separation (∼18 Å), the Coulombic interaction can be approximated as

$$\frac{q\left(F^{\prime }\right)q\left(F\right)}{R}+\frac{q\left(F\right)q\left({\left[4Fe-4S\right]}_{H}\right)}{R}+\frac{q\left(F^{\prime }\right)q\left({\left[4Fe-4S\right]}_{H}\right)}{2R}$$

where *q* represents the formal charge of each Fe–S cluster (−2 for oxidized and −3 for reduced forms, respectively) and R denotes the distance between the F′–F or F–H-clusters. For the first reduction this expression evaluates to +13/R for the **F′**_**red**_ **F**_**ox**_[4Fe–4S]_H_,_ox_ and +14/R for **F′**_**ox**_ **F**_**red**_[4Fe–4S]_H_,_ox_. This difference suggests that the former redox state is energetically more favorable than the latter, owing to reduced Coulombic repulsion. This conclusion is consistent with both experimental observations and the QM/MM calculations.

A similar reasoning applies to the subsequent reduction of **F′**_**red**_ **F**_**ox**_ **H**_**ox**_. In this case, reduction of the [4Fe–4S] cubane within the H-cluster decreases the electrostatic repulsion between electrons, yielding +16.5/R for **F′**_**red**_ **F**_**ox**_[4Fe–4S]_H,red_ and +18/R for **F′**_**red**_ **F**_**red**_[4Fe–4S]_H,ox_. This trend is in agreement with the QM/MM results; however, it does not fully align with experimental data on the catalytic site with PDT instead of ADT,^32^ which indicate that the two states differ by approximately 25 mV, corresponding to only 0.6 kcal/mol in favor of **F′**_**red**_ **F**_**red**_[4Fe–4S]_H,ox_. This small discrepancy between the calculated and experimental values does not appear to be particularly significant. Energy differences of this magnitude are generally at the limit of reliability for DFT methods, as even minor variations in intercluster distances or subtle charge redistribution effects can readily shift the relative stability toward one species or the other. Furthermore, the reported standard reduction potentials were measured for a slightly modified system (PDT rather than ADT on the binuclear cluster), which may plausibly influence the relative stability of the two electromers.

In general, we observed that protonation of the H-cluster promotes electron transfer from F′ to the active site, leading to formation of a species electronically consistent with the **H**_**red**_ **H** ^**+**^ state. At this stage, hydride formation at the binuclear site (**H**_**hyd/ox**_) is favored over protonation at the ADT bridgehead by 14.8 kcal/mol, supporting operation of a proton relay toward the distal iron center. A similar trend is observed upon further reduction: addition of a third electron gives **H**_**sred**_ **H** ^**+**^, and formation of a terminal hydride (**H**_**hyd**_) remains favored over ADT protonation by 19 kcal/mol.

A delicate situation arises for the **F′**_**red**_ **F**_**ox**_ **H**_**red**_ **H** ^**+**^ state. Depending on whether a QM/MM or cluster-based description is adopted, and on how environmental electrostatics are treated, the [4Fe–4S]_H_ subcluster may appear either oxidized or reduced, leading to alternative Fe­(I)­Fe­(I) or Fe­(I)­Fe­(II) descriptions of the diiron site. This near-degeneracy indicates that **H**_**red**_ **H** ^**+**^ occupies a delicate region of the electronic landscape. Consequently, transitions from this state toward either hydride formation or further reduction should be interpreted with caution, even though within our QM/MM framework hydride formation is thermodynamically preferred by ∼15 kcal/mol.

Further protonation at the ADT moiety enables proton transfer to the terminal hydride, a strongly exergonic process that leads to H_2_ formation with essentially no energetic penalty and completes the catalytic sequence.

We note that, in almost all intermediates described above, the F′ cluster remains reduced (Figure 4). In our static representation of the system, the first incoming electron preferentially localizes on F′, such that accumulation of two electrons at the H-cluster requires the addition of three electrons in total. This description does not necessarily reflect the dynamic distribution of electrons under catalytic turnover (where F′ is not expected to act as a permanent electron sink) but rather corresponds to the thermodynamically preferred electron localization within our computational framework. Nevertheless, this analysis provides valuable insight into the intrinsic thermodynamic preferences for both reduction and protonation sites within the enzyme, thereby contributing to a clearer mechanistic picture of the catalytic landscape.

## Conclusions

Our QM/MM investigation of DdHydAB defines a consistent thermodynamic framework for the redox and protonation states accessible along the catalytic cycle. The calculated electron distribution reveals a clear hierarchy within the Fe–S cluster network, highlighting the functional coupling between the accessory clusters and the H-cluster in governing electron flow.

Protonation events are intrinsically linked to electronic redistribution, reinforcing the central role of PCET in [FeFe]-hydrogenases catalysis. While t-H formation at the diiron site represents the productive pathway toward H_2_ evolution, alternative protonation patterns may stabilize electronically favorable yet catalytically inactive configurations, emphasizing the importance of controlled proton delivery.

A key insight of this study is the pronounced sensitivity of intermediate reduction levels (particularly the **H**_**red**_ **H** ^+^ state) to the treatment of the surrounding environment. The presence of near-degenerate electronic configurations underlines the necessity of careful methodological choices when modeling multicluster redox enzymes.

Concluding, this study highlights the importance of explicitly including all accessory Fe–S clusters when modeling electron transfer processes in [FeFe]-hydrogenases, as their presence significantly shapes the electronic distribution and catalytic mechanism. Future investigations should extend this approach to other [FeFe]-hydrogenases, including their full complement of Fe–S clusters, in order to assess the generality and variability of accessory-cluster effects on catalysis. Our results also reinforce that QM/MM remains the method of choice for accurately describing these complex multicluster systems, providing reliable insights into large and electronically intricate enzymes where classical cluster models cannot fully capture their complexity.

## Computational Details

### Protein Setup

All calculations were based on the 1.6 Å resolution crystal structure of the Fe-only hydrogenase from *D. desulfuricans* (PDB ID: 1HFE).^20,71^ The enzyme is a heterodimer composed of a small and of a large subunit. The latter contains the active site and the two accessory [4Fe–4S] clusters. The setup of the protein was the same as in our previous QM/MM studies of [FeFe]-hydrogenases.^48,50,72^

### QM/MM Calculations

All QM/MM calculations were performed using the ComQum software.^73,74^ In such a hybrid approach, the solvated protein is divided into two subsystems: system 1 contains the H, F and F′ clusters. It is treated at QM level and the geometry is allowed to relax. System 2 is composed of the remaining portion of the protein and the surrounding water molecules. It is kept fixed at the crystallographic coordinates and is treated at MM level. Covalent bonds between the QM and the MM regions were treated by the hydrogen link-atom approach,^75^ i.e. the QM system is capped with hydrogen atoms (hydrogen link atoms, HL) whose positions are linearly related to the corresponding carbon atoms (carbon link atoms, CL) in the full system.^73^ The total QM/MM energy in ComQum is calculated as

$$E_{QM/MM}=E_{QM1+ptch2}^{HL}+E_{MM12,q_{1}=0}^{CL}-E_{MM1,q_{1}=0}^{HL}$$

where *E*_QM1+ptch2_^HL^ is the QM energy of System 1 truncated by HL atoms and embedded in the set of point charges modeling System 2. $E_{MM1,q_{1}=0}^{HL}$ is the MM energy of System 1, still truncated by HL atoms and with all charges zeroed. $E_{MM12,q_{1}=0}^{CL}$ is the MM energy of the whole system with CL atoms and with the charges of System 1 set to zero in order to avoid double-counting of the electrostatic interactions.

The QM calculations were performed within the framework of density functional theory (DFT) at the B3LYP/TZVP^76,77^ level of theory using the TURBOMOLE 7.2 software.^78^

For the MM calculations, the Amber software was used^79,80^ The Amber FF14SB force field was employed for the protein^81^ while the general Amber force field^82^ with restrained electrostatic potential (RESP) charges was used for the irons and the ligands.^83^

The QM system (System 1, Figure S1) of the various [FeFe]-hydrogenases models includes the iron and sulfide ions of the H-cluster and of the [4Fe–4S] F– and F′-clusters, the ADT ligand, three CO groups, two CN^–^ ligands, and 12 CH_3_S^–^ fragments that represent the cysteine residues connecting the H, F, and F′-clusters to the rest of the enzyme. Moreover, additional CO, H_2_, SH^–^ or H_2_S molecules were considered in alternative models. The total number of atoms in the QM system was 106 for the resting state enzyme (see Figure S1). The QM region of the model was expanded for **H**_**red**_ **H** ^**+**^ by including six side chains of residues that directly interact with the cyanide ligands, as well as the cysteine residue that interacts with the ADT cofactor (169 atoms, see Figure S3).

The electronic structure of the FeS assemblies was treated by means of the broken-symmetry (BS) approach.^84,85^ All Fe ions are in their high-spin states, but these spins are coupled antiferromagnetically to a lower spin state. The BS coupling is shown in Figure S2.

The various BS states were obtained by swapping the coordinates of the Fe ions, as described in ref 86. In order to test different redox states of the three clusters we enforced different redox states on the subclusters and then we compared the resulting energies of the various possibilities.

An accurate assignment of the H-cluster redox states is an essential premise for the exploration of the electronic structure of the Fe–S clusters of [FeFe]-hydrogenases. To calibrate our theoretical scheme, we compare our QM/MM redox state assignment and equilibrium geometries to those reported for two fully consolidated states: **H**_**ox**_ **–CO** and **H**_**inact**_, the overoxidized inactive state bearing a bound sulfide anion SH^–^ at Fe_d_. For both these states, crystallographic structures and the spectroscopic characterization of the redox states are available.^41,57,87–96^

On the basis of this calibration (see Tables S3 and S4 and Figure S4 in Supporting Information), the QM/MM level of theory employed in the present study was found to be in excellent agreement with both the experimental observations and previously reported DFT results, thus substantiating the reliability of the adopted computational approach. For **H**_**inact**_, we considered the two BS solutions previously reported in ref 48 on FeFe hydrogenase H_inact_, which differ only marginally in energy (0.06 kcal/mol) and spin populations (∼0.01 e^–^).

## Supplementary Material



## Notes

Additional QM/MM and DFT computational results, Mulliken spin populations and charges, optimized geometries, broken-symmetry coupling schemes, electronic-structure analyses, calibration of H_ox_–CO and H_inact_ states, QM/MM calculation for alternative H_hyd_H^+^ state and TD-DFT calculation alternative H_red_ state (PDF)
