# Supplementary material for: QM/MM Modeling of the Electronic Structure and Properties of the Fe–S Clusters in Desulfovibrio desulfuricans [FeFe]-Hydrogenase
Source: Inorg Chem. 2026 Jun 14;65(25):13988–99. doi: 10.1021/acs.inorgchem.6c01252 (PMC13321311; doi:10.1021/acs.inorgchem.6c01252)
Supplement: Supplementary file 1 [file ic6c01252_si_001.pdf]

Supplementary Information - QM/MM modelling of the electronic structure and properties of Fe-S clusters in *Desulfovibrio desulfuricans* FeFe hydrogenases.

Anna Rovaletti,[a,*] Meritxell Wu Lu,[b] Federica Arrigoni,[c] Luca De Gioia,[c] Ulf Ryde,[d] Claudio Greco, Luca Bertini,[c,*]

[a] Department of Earth and Environmental Sciences, University of Milano-Bicocca, Piazza della Scienza 1, 20126 Milan, Italy. anna.rovaletti@unimib.it
[b] Department of Chemistry, Technical University of Berlin, Germany
[c] Department of Biotechnologies and Biosciences, University of Milano-Bicocca, Piazza della Scienza 2, 20126 Milan, Italy, luca.bertini@unimib.it
[d] Department of Theoretical Chemistry, Lund University, Chemical Centre, P.O. Box 124, SE-221 00 Lund, Sweden

Table S1. Fe_P–Fe_D distance in Å, Mulliken spin populations and charges (in brackets) and energy difference in kcal/mol of the various clusters and subclusters of models showing the BS state shown in Figure S2. Atoms belonging to each cluster are shown in Figure S1.

	Fe–Fe distance (Å)	Fe_P	Fe_D	$[2Fe]_H$	$[4Fe4S]_H$	F–cluster	F'–cluster	ΔE (kcal/mol)
**$F'_{ox}F_{ox}H_{ox}$**	2.571	0.16 (-0.30)	1.01 (-0.34)	1.03 (-1.34)	0.01 (-1.65)	0.01 (-2.01)	0.00 (-2.01)	
**$F'_{red}F_{ox}H_{ox}$ (+1e$^-$)**	2.566	0.14 (-0.30)	0.98 (-0.35)	1.01 (-1.35)	0.02 (-1.64)	-0.01 (-2.01)	-0.98 (-3.00)	
**$F'_{red}F_{ox}H_{red}$**	2.570	0.14 (-0.31)	1.01 (-0.36)	1.02 (-1.46)	-0.97 (-2.52)	0.02 (-2.02)	-0.98 (-3.00)	
**$F'_{red}F_{red}H_{red}$**	2.570	0.14 (-0.31)	1.02 (-0.36)	1.02 (-1.49)	-0.97 (-2.50)	0.97 (-3.01)	0.98 (-3.01)	
**$H_{ox}H^+$//N(ADT)**	2.575	0.08 (-0.35)	1.04 (-0.36)	1.00 (-0.62)	-0.93 (-2.35)	0.00 (-2.01)	-0.02 (-2.02)	0.0

$F'_{red}F_{ox}H_{ox} + H^+$								
	Fe–Fe distance (Å)	Fe_P	Fe_D	$[2Fe]_H$	$[4Fe4S]_H$	F–cluster	F'–cluster	ΔE (kcal/mol)
**H^+@S1**	2.569	0.14 (-0.34)	0.97 (-0.35)	1.01 (-1.14)	-0.96 (-1.84)	0.00 (-2.01)	0.00 (-2.01)	5.6
**H^+@S2**	2.571	0.15 (-0.29)	1.01 (-0.35)	1.02 (-1.29)	-0.97 (-1.69)	0.00 (-2.01)	0.00 (-2.01)	9.0
**H^+@S3**	2.569	0.15 (-0.30)	1.01 (-0.35)	1.03 (-1.27)	-0.98 (-1.73)	0.01 (-2.00)	0.00 (-2.00)	0.4
**H^+@S4**	2.573	0.15 (-0.31)	1.00 (-0.34)	1.02 (-1.27)	-0.95 (-1.72)	0.00 (-2.01)	0.00 (-2.01)	6.2
**H^+@μH**	2.590	0.02 (-0.29)	0.00 (-0.52)	0.02 (-1.34)	-0.01 (-1.65)	0.00 (-2.01)	0.00 (-2.01)	-25.9
**H^+@FeD**	2.545	0.01 (-0.30)	0.00 (-0.58)	0.01 (-1.36)	0.00 (-1.62)	0.00 (-2.01)	0.00 (-2.01)	-12.4
**H^+@tH**	2.607	0.02 (-0.19)	0.00 (-0.57)	0.02 (-1.33)	-0.01 (-1.61)	0.00 (-2.01)	0.00 (-2.01)	-3.00
**$F'_{red}F_{ox}H_{red} + H^+$**								
**H^+@N(adt)**	2.576	0.08 (-0.35)	1.04 (-0.37)	0.98 (-0.63)	-0.94 (-2.35)	0.01 (-2.02)	0.98 (-3.00)	0.0
**H^+@S1**	2.570	0.14 (-0.34)	0.97 (-0.35)	1.01 (-1.15)	-0.96 (-1.83)	0.01 (-2.02)	0.98 (-3.00)	4.9
**H^+@S2**	2.571	0.15 (-0.29)	1.01 (-0.35)	1.02 (-1.30)	-0.97 (-1.69)	0.01 (-2.02)	0.98 (-3.00)	7.3
**H^+@S3**	2.570	0.15 (-0.30)	1.01 (-0.35)	1.03 (-1.27)	-0.98 (-1.73)	0.01 (-2.01)	0.98 (-3.00)	-3.9
**H^+@S4**	2.572	0.15 (-0.31)	1.00 (-0.35)	1.02 (-1.28)	-0.95 (-1.71)	0.01 (-2.02)	0.98 (-3.00)	4.0
**H^+@μH**	2.590	0.02 (-0.29)	0.00 (-0.52)	0.01 (-1.35)	-0.01 (-1.64)	0.00 (-2.01)	0.98 (-3.00)	-28.0
**H^+@FeD**	2.545	0.01 (-0.30)	0.00 (-0.58)	0.01 (-1.37)	0.00 (-1.62)	0.00 (-2.01)	0.98 (-3.00)	-14.8

	Fe–Fe distance (Å)	Fe_P	Fe_D	$[2Fe]_H$	$[4Fe4S]_H$	F–cluster	F'–cluster	ΔE (kcal/mol)
**H^+@tH**	2.609	0.02 (-0.19)	0.00 (-0.57)	0.02 (-1.39)	-0.01 (-1.60)	0.01 (-2.01)	0.98 (-3.00)	-5.4
**$H_{red}H^+$ H^+@N(adt) sp PBE0**	2.576	0.09 (-0.41)	1.08 (-0.41)	0.99 (-0.60)	-0.95 (-2.39)	0.00 (-2.01)	0.00 (-2.01)	
**$H_{red}H^+$ H^+@N(adt) (sp TPSSh**	2.576	-0.04 (-0.39)	0.93 (-0.40)	0.80 (-0.74)	-0.76 (-2.25)	0.00 (-2.01)	0.00 (-2.01)	
**$H_{red}H^+$ H^+@N(adt) (sp M06**	2.576	0.05 (-0.18)	1.14 (-0.27)	0.98(-0.64)	-0.94 (-2.34)	0.01 (-2.02)	0.97 (-3.00)	
**$F'_{red}F_{ox}H_{sred}H^+$**	2.610	-0.04 (-0.27)	0.11 (-0.47)	0.07 (-1.35)	-0.97 (-2.57)	-0.06 (-2.08)	0.98 (-3.00)	
**$F'_{red}F_{ox}H_{hyd}$**	2.548	-0.01 (-0.32)	0.00 (-0.58)	-0.01 (-1.49)	-0.97 (-2.49)	0.00 (-2.02)	0.98 (-3.00)	
**$F'_{red}F_{ox}H_{hyd:ox}$**	2.545	0.01 (-0.30)	0.00 (-0.58)	0.01 (-1.37)	0.00 (-1.62)	0.00 (-2.01)	0.98 (-3.00)	
**$F'_{red}F_{ox}H_{hyd}H^+$**	2.542	0.00 (-0.57)	-0.03 (-0.37)	-0.03 (-0.69)	1.02 (-2.30)	0.00 (-2.02)	-0.99 (-3.00)	
**$F'_{red}F_{ox}$**$[4Fe\text{-}4S]_{H,red}$Fe(II) Fe(II)H^-	2.617	-0.70 (-0.24)	-0.21 (-0.48)	-0.90 (-1.17)	-0.10 (-1.82)	0.01 (-2.01)	0.98 (-3.00)	
**$F'_{ox}F_{ox}H_{hyd:ox}$**	2.542	-0.02 (-0.37)	0.00 (-0.57)	-0.02 (-0.67)	1.01 (-2.31)	0.00 (-2.01)	0.00 (-2.01)	
**$F'_{red}F_{ox}H_{ox}H_2$**	2.624	-0.43 (-0.30)	-0.48(-0.44)	-0.95 (-1.29)	-0.07 (-1.70)	0.01 (-2.01)	0.98 (-3.00)	

Table S2. Fe_P–Fe_D distance in Å, Mulliken spin populations and charges of the various clusters and subclusters of models showing the BS state shown in Figure S2. QM systems of different sizes were employed: the one employed in most of the present work shown in Figure S1 and an enlarged QM system – referred in the Table as “with residues” – shown in Figure S3.

			**Charges**				**Spin**			
		**Fe_p-Fe_d**	**2Fe**	**[4Fe-4S]**	**F**	**F’**	**2Fe**	**[4Fe-4S]**	**F**	**F’**
QM/MM	**$F'_{ox}F_{ox}H_{ox}$**	2.57	-1.34	-1.65	-2.01	-2.01	1.05	0.01	0.01	0.00
QM/MM	**$F'_{red}F_{ox}H_{ox}$**	2.57	-1.35	-1.64	-2.01	-3.00	-1.04	-0.02	0.01	0.98
QM/MM	**$F'_{red}F_{ox}H_{red}$**	2.57	-1.46	-2.52	-2.02	-3.00	-1.04	0.97	0.02	0.98
QM	**H_{red} ε=4**	2.57	-1.49	-2.49	-2.02	-3.00	-1.17	1.09	0.08	0.96
QM	**H_{red} vacuo**	2.57	-1.57	-2.42	-2.02	-3.00	-1.21	1.10	-0.03	0.97
QM/MM	**H_{red} with residues**	2.57	-0.45	-2.32	-2.23	-3.00	1.01	0.79	0.22	-0.99
QM	**H_{red} with residues ε=4**	2.57	-0.62	-2.32	-2.02	-3.00	1.01	0.79	1.00	-0.99
QM	**H_{red} with residues vacuo**	2.57	-1.6	-2.21	-2.01	-2.16	-0.03	1.18	0,02	-0.15
QM/MM	**$F'_{red}F_{ox}H_{red}H^+$**	2.58	-0.63	-2.35	-2.02	-3.00	0.96	-0.94	0.01	0.98
QM	**$H_{red}H^+$ ε=4**	2.58	-0,62	-2.36	-2.02	-3.00	0.96	-0.86	0.01	0.98
QM	**$H_{red}H^+$ vacuo**	2.58	-1.38	-1.61	-2.01	-3.00	-0.01	-0.03	0.01	0.98
QM/MM	**$H_{red}H^+$ with residues**	2.59	-0.40	-2.38	-2.02	-3.00	0.99	-0.09	0.16	0.98
QM/MM	**$H_{red}H^+$ with**	2.59	-0.40	-1.59	-2.01	-3.00	0.02	-0.04	0.01	0.98

	**residues ε=4**									
QM	**$H_{red}H^+$ with residues vacuo**	2.59	-1.45	-1.54	-2.00	-2.01	0.93	0	0.01	0.01

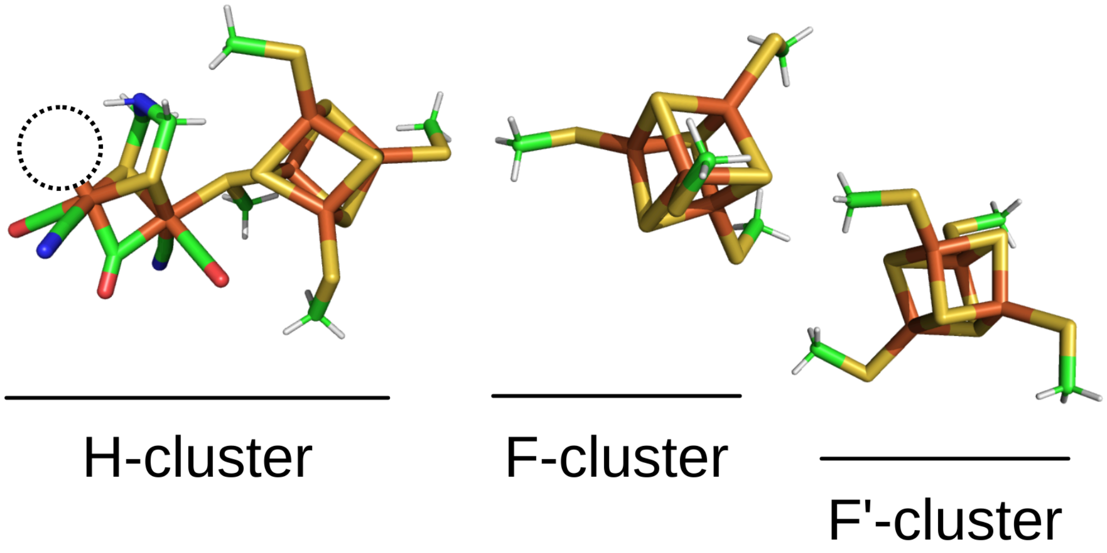

Figure S1. QM system of the QM/MM models. The dashed circle highlights an apical vacancy at distal iron Fe_D at which position we insert a CO, H_2, SH^- or H_2S molecule to create different QM/MM models of [FeFe]-hydrogenases (106 atoms). Color scheme: white, H; green, C; blue, N; red, O; yellow, S; orange, Fe.

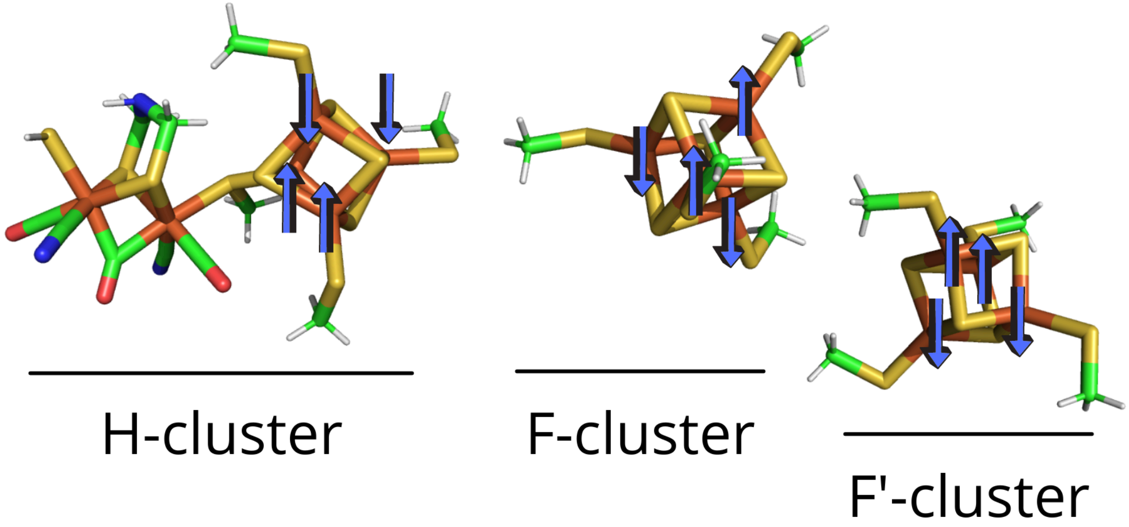

Figure S2. Broken-symmetry coupling scheme used for all the models discussed in the main text of the present paper. Arrows pointing up and down indicate alpha and beta spin excess, respectively. In the BS2 coupling scheme adopted for $\mathbf{H}_{\mathbf{inact}}$ state the $[4Fe\text{-}4S]_H$ are flipped

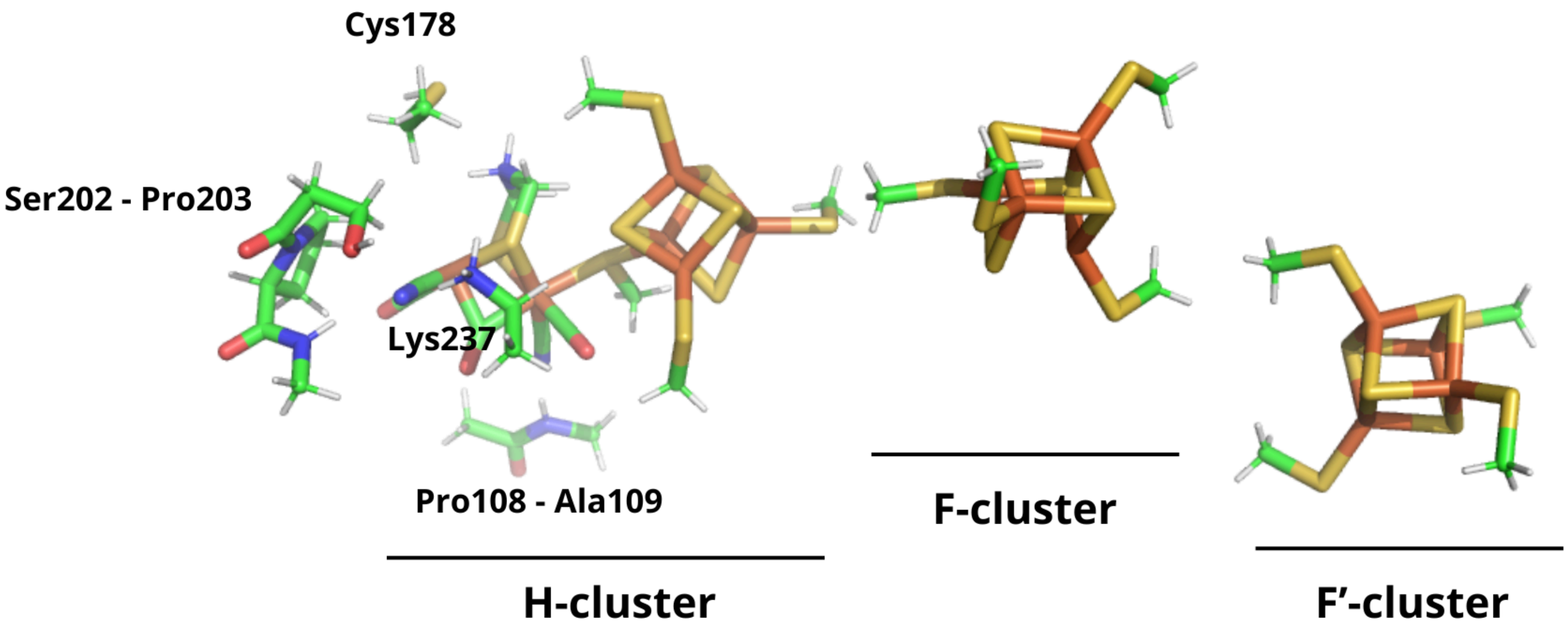

Figure S3. The enlarged QM system of the QM/MM model (169 atoms).

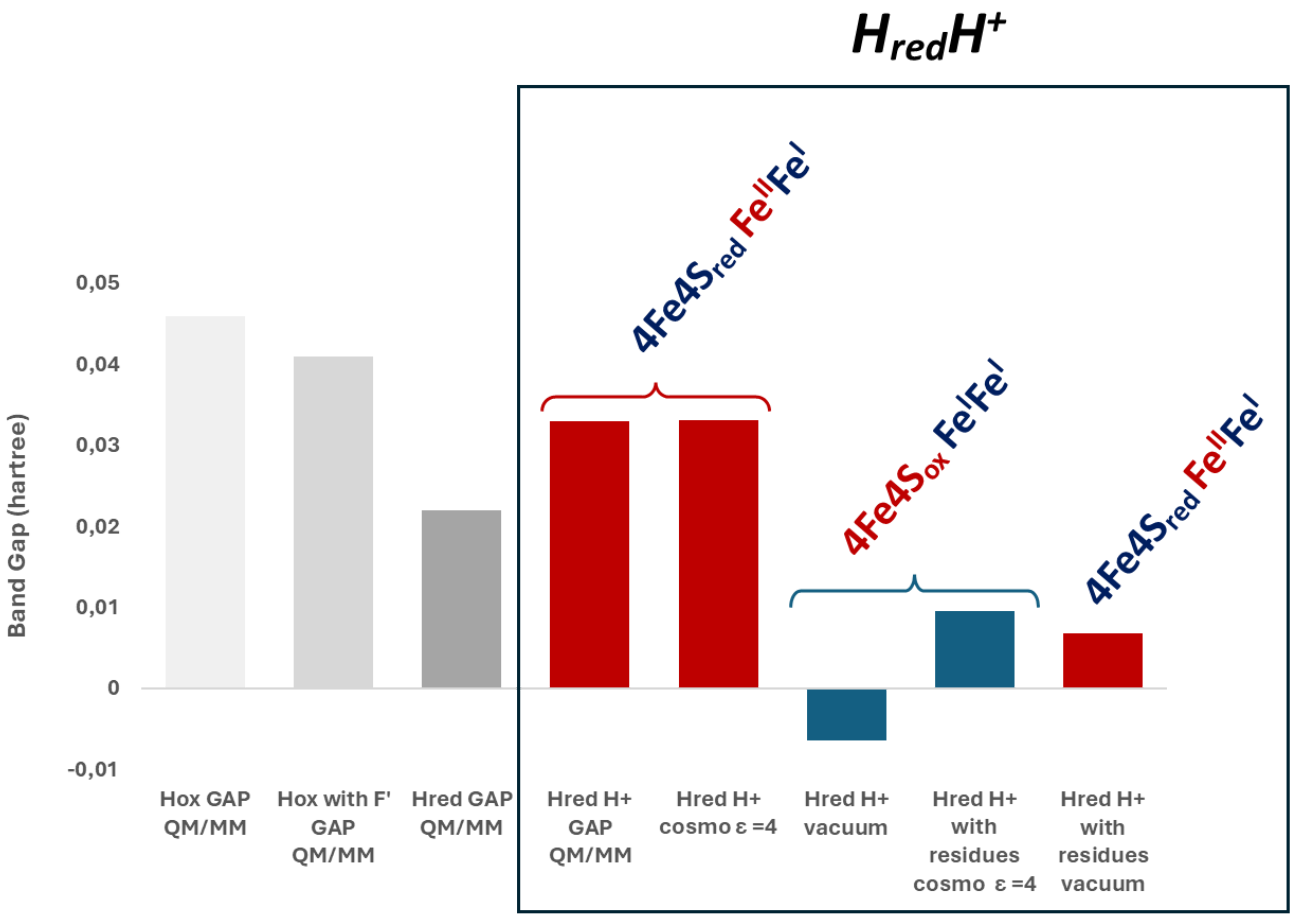

Figure S4. HOMO-LUMO gaps valence MO orbital contributions obtained for **$F'_{ox}F_{ox}H_{ox}$**, **$F'_{red}F_{ox}H_{ox}$**, **$F'_{red}F_{ox}H_{red}$** and **$F'_{red}F_{ox}H_{red}$ + H^+@ADT** using either the QM/MM model with enlarged QM system or QM model in the vacuum or in solvent (dielectric constant set to 4).

H_{ox}-CO and H_{inact} states: calibration of the QM/MM predictions

The accurate assignment of the H-cluster redox states is an essential premise for the exploration of the electronic structure of the Fe-S clusters of [FeFe]-hydrogenases. To calibrate our theoretical scheme, we compare our QM/MM redox state assignment and equilibrium geometries to the ones reported in the case of two selected fully consolidated states: **H_{ox}-CO** and **H_{inact}**. For both these redox states the crystallographic structures and the spectroscopic characterization of the redox states are available.(Duan et al. 2022a; Rodríguez-Maciá et al. 2020a)

CO is a potent inhibitor of FeFe hydrogenases in its resting state **H_{ox}** yielding the formation of the CO-inhibited form (**H_{ox}-CO**). This form has been extensively studied using various techniques. (Popescu and Münck 1999) (Roseboom et al. 2006) (Silakov et al. 2007; Fiedler and Brunold 2005) (Silakov et al. 2009a; Sensi et al. 2016a).(Lemon and Peters 1999a) (Reijerse, Birrell, and Lubitz 2020a)

The electronic structure of **H_{ox}-CO** proposed in the literature is S=1/2 EPR active with the spin density mostly localized around the proximal Fe thus indicating a $Fe(I)_P$-$Fe(II)_D$ redox state for 2Fe.(Silakov et al. 2009b) This result has been recently confirmed by Reijerse *et al.* (Reijerse, Birrell, and Lubitz 2020b) on the basis of ^{13}C electron–nuclear–nuclear triple resonance spectroscopy.

The QM/MM optimized geometry are in very nice agreement with the those of XRD structures, with Fe–Fe distance in $[2Fe]_H$ equal to 2.660 Å (2.598 Å PDB:1C4C (Lemon and Peters 1999b) and 2.595 Å PDB:8ALN, (Duan et al. 2022b) while the Fe–CO_{ex} bond distance is 1.799 Å (1.803 Å PDB:1C4C; 1.790 Å PDB:8ALN), see Table S3. In Figure S4 and Table S4 are reported the electronic structures and the cluster total charges and spin populations of the **H_{ox}-CO** forms. The **$F'_{ox}F_{ox}H_{ox}$-CO** state with the F, F' and $[4Fe4S]_H$ in oxidized state has a spin population on the $2[Fe]_H$ equal to 0.98, with 0.54 on Fe_D and 0.32 on Fe_P (that

corresponds to a ratio of 1.7:1 which must be compared with experimental 5:1. (Silakov et al. 2009c; Sensi et al. 2016b) From these values we can conclude that $[2Fe]_H$ is better described as valence-localized $Fe(I)_P$-$Fe(II)_D$ redox state, also in agreement with DFT assignment by Brunold *et al. (Silakov et al. 2007; Fiedler and Brunold 2005)* on $4Fe4S]_H$-$[2Fe]_H$ model. Upon mono-electronic reduction of **$F'_{ox}F_{ox}H_{ox}$-CO**, we observe the reduction of the F' cluster with the atomic and spin population on 2Fe are almost unaffected by this. In this new F' reduced form, $[2Fe]_H$ Fe–Fe distance is elongated only by 0.002 Å. This result implies that, up to the reduction of F', the two structures can both describe **H_{ox}-CO**.

The FeFe hydrogenase can be purified in an EPR silent inactive state called **H_{inact}** or **$H_{ox.air}$** whose structure and formation mechanism has been elusive for years. This inactive form originally identified in *Desulfovibrio desulfuricans(Hatchikian et al. 1992)* is O_2-stable and can be reactivated upon reduction. Initially identified as an overoxidized form with $[2Fe]_H$ in $Fe(II)_P$-$Fe(II)_D$ redox state with additional ligand bound to Fe_D, (Rodríguez-Maciá et al. 2018) (Rodríguez-Maciá et al. 2020b) (Felbek et al. 2021) **H_{inact}** has recently been characterized as an exogenous sulfide inhibited overoxidized **$H_{overox}SH^-$** form with $[2Fe]_H$ in a $Fe(II)_P$-$Fe(II)_D$ state.

QM/MM confirms that this state is EPR silent, being constituted by a Fe(II)Fe(II) binuclear center and by three oxidized 2Fe(II)2Fe(III) cubanes. The calculated Fe_P–Fe_D and Fe_D–S(SH) distances are 2.519 and 2.289 Å, in good agreement with the XRD values of 2.647 and 2.414 Å in PDB:6SG2 for the *D. desulfurican*s in **H_{inact}** state. This indicates that our method is reliable in reproducing in detail both structural and electronic features of H-cluster states with very different redox properties. When **H_{inact}** and **H_{ox}-CO** are $1e^-$ reduced, we obtain two new states which are reduced at the F' cluster in both cases. This reduction does not imply any significant differences in the minimum geometries and atomic charge and spin distributions when compared to the fully oxidized state (see Table S4).

A further mono-eletronic reduction of **F'$_{red}$F$_{ox}$H$_{ox}$-CO** give rise to the **F'$_{red}$F$_{ox}$H$_{red}$-CO** form which has been proposed by Adamska et al. (Adamska-Venkatesh et al. 2014) (Rodríguez-Maciá et al. 2017) by electrochemical FTIR. According to this study, the structure of **F'$_{red}$F$_{ox}$H$_{red}$-CO** is similar to that of **F'$_{red}$F$_{ox}$H$_{ox}$-CO** but the IR spectrum of the former results red shifted. The electron reduces the [4Fe4S]$_H$ cluster with a **F'$_{red}$F$_{ox}$H$_{ox}$-CO/F'$_{red}$F$_{ox}$H$_{red}$-CO** potential of -570 mV while the [2Fe]$_H$ maintains the Fe(I)$_P$-Fe(**II**)$_D$ redox state. QM/MM nicely confirms the experimental data, finding that, beside on F', the spin population on [4Fe4S]$_H$ and on the Fe$_D$ and Fe$_P$ is almost identical to that of **F'$_{red}$F$_{ox}$H$_{ox}$-CO** (see Table S4).

Table S3. XRD distances of the $[2Fe]_H$ in *Clostridium pasteurianum* (PDB:8ALN) and in QM/MM optimized geometries. All distances in Å.

	PDB:8ALN	$\mathbf{H_{ox}}$**-CO**	$\mathbf{H_{ox}}$**-CO (+1e$^-$)**	$\mathbf{H_{ox}}$**-CO (+2e$^-$)**
Fe_P-Fe_D	2.595	2.660	2.662	2.667
Fe_D-C(COinhib)	1.790	1.779	1.779	1.778
Fe_D-C(μCO)	1.970	2.043	2.047	2.051
Fe_D-C(CO)	1.764	1.797	1.796	1.794
Fe_D-C(CN)	1.945	1.925	1.925	1.925
Fe_P-C(μCO)	1.898	1.903	1.901	1.899
Fe_P-C(CO)	1.725	1.774	1.775	1.777
Fe_P-C(CN)	1.840	1.920	1.919	1.918

Table S4. Fe_P–Fe_D distance in Å, Mulliken spin populations and charges (in brackets) of the various clusters and subclusters of models showing the BS state shown in Figure S2.

	**Fe–Fe distance (Å)**	**Fe_P**	**Fe_D**	**$[2Fe]_H$**	**$[4Fe4S]_H$**	**F–cluster**	**F'–cluster**
**$F'_{ox}F_{ox}H_{ox}$-CO**	2.660	0.54 (-0.30)	0.32 (-0.54)	0.98 (-1.28)	0.05 (-1.71)	0.01 (-2.01)	0.00 (-2.01)
**$F'_{red}F_{ox}H_{ox}$-CO**	2.662	0.52 (-0.30)	0.33 (-0.54)	0.98 (-1.29)	0.06 (-1.70)	0.01 (-2.01)	-0.99 (-3.00)
**$F'_{red}F_{ox}H_{red}$-CO**	2.667	0.50 (-0.31)	0.34 (-0.54)	0.96 (-1.32)	-0.97 (-3.00)	0.06 (-1.68)	0.98 (-3.00)
**$F'_{ox}F_{ox}H_{overox}$-SH^-**	2.519	0.01 (-0.35)	0.00 (-0.51)	0.01 (-1.41)	-0.01 (-1.58)	0.01 (-2.01)	0.00 (-2.01)
**$F'_{red}F_{ox}H_{overox}$-SH^- BS1 state**	2.515	0.01 (-0.36)	0.00 (-0.51)	0.01 (-1.42)	-0.01 (-1.57)	0.01 (-2.01)	0.98 (-3.00)
**$F'_{red}F_{ox}H_{overox}$-SH^- BS2 state**	2.515	-0.01 (-0.36)	0.00 (-0.51)	-0.01 (-1.42)	0.01 (-1.57)	0.00 (-2.01)	0.98 (-3.00)

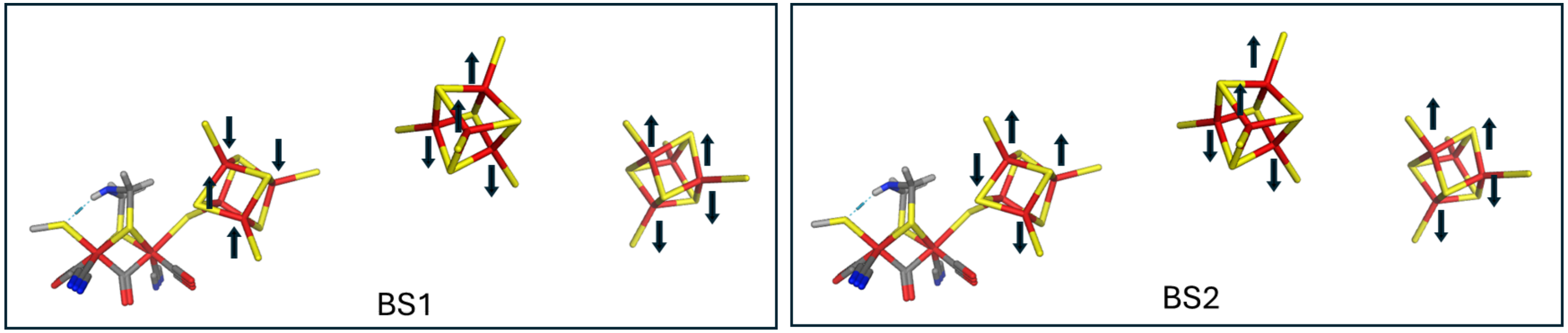

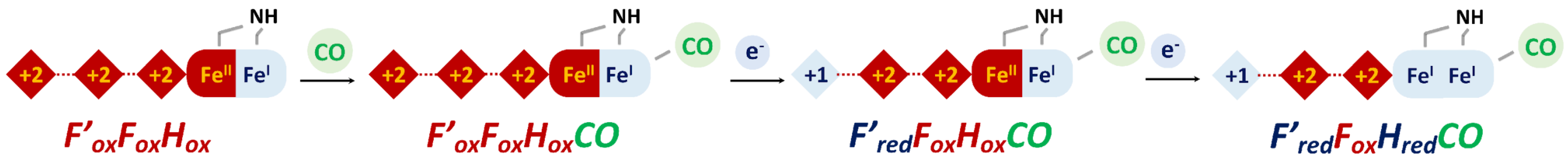

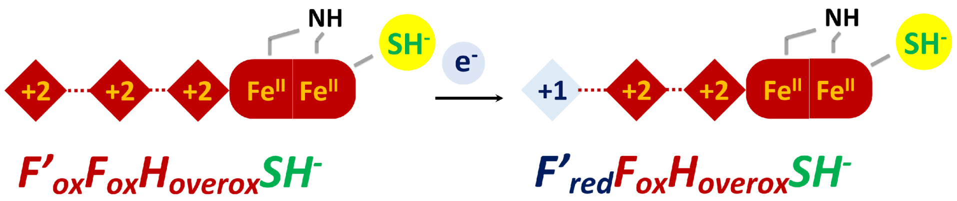

Figure S4. Electronic structure of the **H_{ox}-CO** and **H_{overox}** states. The reduced and oxidized state of the Fe-S clusters are evidenced in blue and orange, respectively. Going from left to right are the F', F, $[4Fe4S]_H$ and $2[Fe]_H$ clusters. On top from the left is the electronic structure of the fully oxidized **H_{ox}-CO** states. Upon its one-electron reduction, F' is reduced with negligible effect

on $[2Fe]_H$. In both states the $[2Fe]_H$ is in a $Fe(I)_P$-$Fe(II)_D$ redox state. Below the electronic structure of the $\mathbf{H_{overox}}$**-SH**$^-$ ($\mathbf{H_{inact}}$) with $[2Fe]_H$ in a $Fe(II)_P$-$Fe(II)_D$ state (over oxidized state of binuclear cluster).

Detailed analysis of the $\mathbf{H_{red}H^+}$ states. B3LYP, PBE0, TPSSh and M06 all yield the same Fe(I)Fe(II) state (see Table S1). We explored alternative initial structures to assess their influence on the resulting electronic configuration. In particular we started from either the more reduced $\mathbf{F'_{red}F_{ox}H_{sred}H^+}$ species or the more oxidized $\mathbf{F'_{red}F_{ox}H_{ox}H^+}$ species at their equilibrium geometries, removing or adding one electron without relaxing the geometry. In both cases during the first SCF calculation, the electron is spontaneously transferred to the $[4Fe\text{-}4S]_H$ cluster.

A further attempt in this direction was made by noticing that the $[4Fe\text{-}4S]_{H,ox}$-Fe(I)Fe(I) $\mathbf{F'_{red}F_{ox}H_{red}H^+}$ state is an electronic excited state of the $\mathbf{F'_{red}F_{ox}H_{red}H^+}$ $[4Fe\text{-}4S]_{red}$-Fe(I)Fe(II) state. The electronic structure of this state in DdHydAB has previously been investigated in our laboratory using a cluster model only including the H-cluster, obtaining $[4Fe\text{-}4S]_{H,ox}$-Fe(I)Fe(I) electronic structure. Within this model, we computed the first 20 open-shell singlet excited states at the TD-DFT level. Analysis of molecular orbital populations reveals that the fifteenth excited state corresponds to a Fe(I)Fe(I) → $[4Fe\text{-}4S]_{H,ox}$ charge transfer yielding an electronically excited configuration with a reduced cubane and a Fe(I)Fe(II) diiron center. Unfortunately, we were unable to compute the excited states, because the QM/MM TD-DFT calculations did not converge. Therefore, we could not determine the energy of the true $\mathbf{F'_{red}F_{ox}H_{red}H^+}$ state featuring a reduced di-iron center.

A further attempt started from the observation that the protonated ADT could be stabilized by a H-bond to the Cys169 sulfide group. Therefore, we enlarged the QM portion of the QM/MM model including the Cys169 residue, but also this model gave a $[4Fe\text{-}4S]_{H,red}$-Fe(I)Fe(II) state (Table S2).

None of the approaches considered above yields the previously reported electronic structure. We then examined whether the discrepancy could be related to the specific protein configuration used in the QM/MM setup, which had originally been constructed based on the **F’$_{ox}$F$_{ox}$H$_{ox}$** state.

We therefore considered a series of calculations in which the protein environment was removed, performing DFT calculations. Two cases were examined: in the first, the Fe–S clusters were left 'bare', surrounded only by the cysteine residues necessary to maintain their crystallographic positions; in the second, we included six residues (Cys177, Ser201, Pro107, Ala108. Pro202) that are close to the H-cluster, supporting proper coordination of the cyanide ligands ions (Figure S3). In addition, two different levels of theory were employed, involving vacuum calculations or an inclusion of environmental effects via continuum solvation (COSMO, $\varepsilon=4$).

The most relevant result emerging from these calculations is that the expected electronic configuration — with the binuclear cluster in the reduced Fe(I)Fe(I) state and the [4Fe–4S]$_H$ cubane oxidized — is obtained either in the vacuum model without additional residues or in the extended model with COSMO solvation. To verify this result, we also performed cross-tests: COSMO calculations were started from the vacuum-optimized wavefunction, and vacuum calculations were started from the COSMO-optimized wavefunction, consistently converging to the same electronic state.

Analysis of the optimized geometries for the various **F’$_{red}$F$_{ox}$H$_{red}$H$^+$** models reveals minimal structural variations, indicating that the core geometry does not change significantly across the different computational setups. However, despite this geometric consistency, the electronic structure proves to be sensitive to the model and DFT level. Examination of the HOMO-LUMO gaps shows noticeable variations, suggesting that these electronic properties are influenced by the specific type of model used and the methodology employed to account for solvent and dispersion effects (Figure S4).

To verify the consistency of the calculations performed on the $\mathbf{F'_{red}F_{ox}H_{red}H^+}$ species in the absence of the protein frame (i.e., without the QM/MM level of theory), we applied the same approach to the $\mathbf{F'_{red}F_{ox}H_{red}}$ species. In all four cases examined, we systematically observe the same electronic structure as discussed above, characterized by a reduced $[4Fe\text{-}4S]_H$ cluster and a binuclear site in the Fe(I)Fe(II) redox state (see Table S2).

$\mathbf{H_{hyd}H^+}$ states at QM/MM level and $\mathbf{H_{red}}$ state at TD-DFT QM/MM level

The only case in which the QM/MM calculations converged to more than one self-consistent solution, distinct from the global minimum-energy state ($\mathbf{F'_{red}F_{ox}H_{hyd}H^+}$), was the $\mathbf{H_{hyd}H^+}$ state. In this case, two converged electronic solutions were obtained. The first corresponds to the lowest-energy state, characterized by an oxidized binuclear subcluster, a hydride bound to the distal Fe center, a protonated ADT bridgehead, and a reduced [4Fe–4S] cubane of the H-cluster. A second solution was also identified, in which the proximal Fe center is reduced at the expense of oxidation of the cubane subcluster. These two states therefore represent two electromers of the same chemical system. Their equilibrium geometries are highly similar, while the second electronic state lies 7 kcal mol^{-1} higher in energy.

Remarkably, for both electronic solutions, bringing the hydride bound to the distal Fe center closer to the proton on the ADT moiety leads the system to converge to the same $\mathbf{F'_{red}F_{ox}H_{ox}H_2}$ state.

We estimate at TD-DFT level the energy differences between $\mathbf{H_{red}}$ states characterized by different oxidation patterns of the accessory clusters. At the QM/MM level, we were unable to converge the wavefunction to any electronic configuration other than the ground state, even when starting from initial guesses specifically constructed to impose the desired oxidation state distribution. In all such cases, the SCF procedure invariably relaxed to the lowest-energy electronic solution, corresponding to the

ground-state oxidation pattern of the clusters, indicating that alternative frontier-MO occupations cannot be stabilized as self-consistent solutions within our computational framework. To probe the energetic cost associated with such alternative arrangements, we therefore considered a hypothetical $\mathbf{H}_{\mathbf{red}}$ state with both F′ and F reduced and an oxidized cubane as an electronically excited state of the ground-state $\mathbf{H}_{\mathbf{red}}$ configuration. Time-dependent DFT calculations place this state 7.6 kcal mol^{-1} above the ground state. Although this value provides a qualitative estimate of the energetic penalty associated with this oxidation-state redistribution, a direct comparison with experiment is not straightforward, as this transition is not directly accessible by UV–vis spectroscopy.

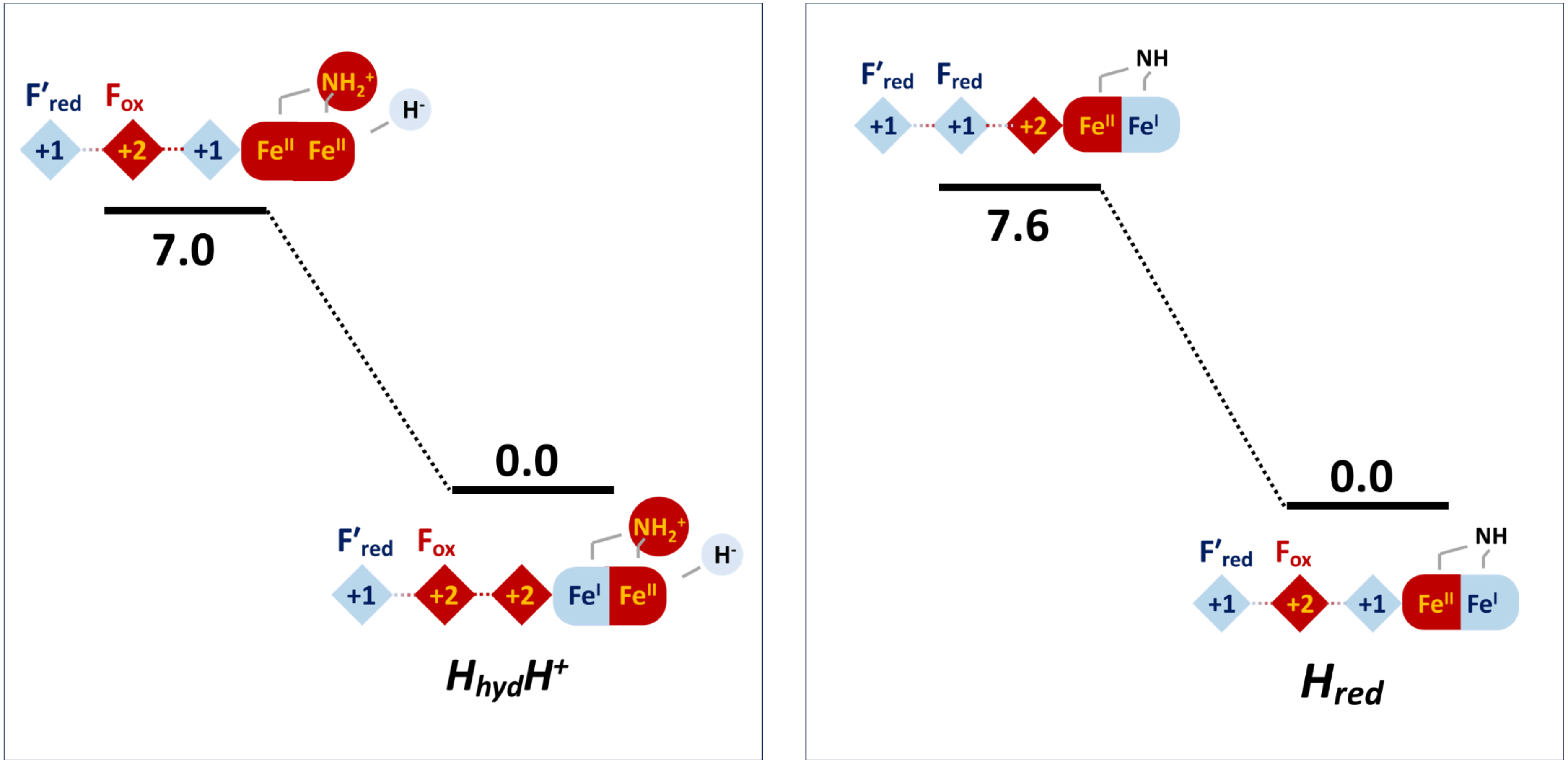

Figure S5. On the left: **QM/MM** energy difference between **$F'_{red}F_{ox}H_{hyd}H^{+}$** and **$F'_{red}F_{red}$**$[4Fe\text{-}4S]_{H,ox}Fe(I)Fe(II)H^{-}$ states. On the right: TD-DFT QM/MM energy difference between two **H_{red}** states i.e **$F'_{red}F_{ox}$**$[4Fe\text{-}4S]_{H,red}$ and **$F'_{red}F_{red}$**$[4Fe\text{-}4S]_{H,ox}$ being the latter the lowest ground **H_{red}** state state. Energy in kcal/mol.

Adamska-Venkatesh, Agnieszka, Danuta Krawietz, Judith Siebel, Katharina Weber, Thomas Happe, Edward Reijerse, and Wolfgang Lubitz. 2014. "New Redox States Observed in [FeFe] Hydrogenases Reveal Redox Coupling within the H-Cluster." *Journal of the American Chemical Society* 136 (32): 11339–46.

Duan, Jifu, Anja Hemschemeier, David J. Burr, Sven T. Stripp, Eckhard Hofmann, and Thomas Happe. 2022a. "Cyanide Binding to [FeFe]-Hydrogenase Stabilizes the Alternative Configuration of the Proton Transfer Pathway." *Angewandte Chemie* , December. https://doi.org/10.1002/anie.202216903.

Felbek, Christina, Federica Arrigoni, David de Sancho, Aurore Jacq-Bailly, Robert B. Best, Vincent Fourmond, Luca Bertini, and Christophe Léger. 2021. "Mechanism of Hydrogen Sulfide-Dependent Inhibition of FeFe Hydrogenase." *ACS Catalysis*. https://doi.org/10.1021/acscatal.1c04838.

Fiedler, Adam T., and Thomas C. Brunold. 2005. "Computational Studies of the H-Cluster of Fe-Only Hydrogenases: Geometric, Electronic, and Magnetic Properties and Their Dependence on the [Fe_4S_4] Cubane." *Inorganic Chemistry*. https://doi.org/10.1021/ic050946f.

Hatchikian, E. C., N. Forget, V. M. Fernandez, R. Williams, and R. Cammack. 1992. "Further Characterization of the [Fe]-Hydrogenase from Desulfovibrio Desulfuricans ATCC 7757." *European Journal of Biochemistry / FEBS* 209 (1): 357–65.

Lemon, B. J., and J. W. Peters. 1999a. "Binding of Exogenously Added Carbon Monoxide at the Active Site of the Iron-Only Hydrogenase (CpI) from Clostridium Pasteurianum." *Biochemistry* 38 (40): 12969–73.

Popescu, Codrina V., and Eckard Münck. 1999. "Electronic Structure of the H Cluster in [Fe]-Hydrogenases." *Journal of the American Chemical Society*. https://doi.org/10.1021/ja991243y.

Reijerse, Edward, James A. Birrell, and Wolfgang Lubitz. 2020a. "Spin Polarization Reveals the Coordination Geometry of the [FeFe] Hydrogenase Active Site in Its CO-Inhibited State." *Journal of Physical Chemistry Letters* 11 (12): 4597–4602.

Rodríguez-Maciá, Patricia, Lisa M. Galle, Ragnar Bjornsson, Christian Lorent, Ingo Zebger, Yoshitaka Yoda, Stephen P. Cramer, Serena DeBeer, Ingrid Span, and James A. Birrell. 2020a. "Caught in the H_{inact} : Crystal Structure and Spectroscopy Reveal a Sulfur Bound to the Active Site of an O_2 -stable State of [FeFe] Hydrogenase." *Angewandte Chemie International Edition*. https://doi.org/10.1002/anie.202005208.

Rodríguez-Maciá, Patricia, Edward J. Reijerse, Maurice van Gastel, Serena DeBeer, Wolfgang Lubitz, Olaf Rüdiger, and James A. Birrell. 2018. "Sulfide Protects [FeFe] Hydrogenases From O." *Journal of the American Chemical Society* 140 (30): 9346–50.

Rodríguez-Maciá, Patricia, Edward Reijerse, Wolfgang Lubitz, James A. Birrell, and Olaf Rüdiger. 2017. "Spectroscopic Evidence of Reversible Disassembly of the [FeFe] Hydrogenase Active Site." *Journal of Physical Chemistry Letters* 8 (16): 3834–39.

Roseboom, Winfried, Antonio L. De Lacey, Victor M. Fernandez, E. Claude Hatchikian, and Simon P. J. Albracht. 2006. "The Active Site of the [FeFe]-Hydrogenase from Desulfovibrio Desulfuricans. II. Redox Properties, Light Sensitivity and CO-Ligand Exchange as Observed by Infrared Spectroscopy." *Journal of Biological Inorganic Chemistry: JBIC: A Publication of the Society of Biological Inorganic Chemistry* 11 (1): 102–18.

Sensi, Matteo, Carole Baffert, Claudio Greco, Giorgio Caserta, Charles Gauquelin, Laure Saujet, Marc Fontecave, et al. 2016a. "Reactivity of the Excited States of the H-Cluster of FeFe Hydrogenases." *Journal of the American Chemical Society*. https://doi.org/10.1021/jacs.6b06603.

Silakov, Alexey, Eduard J. Reijerse, Simon P. J. Albracht, E. Claude Hatchikian, and Wolfgang Lubitz. 2007. "The Electronic Structure of the

H-Cluster in the [FeFe]-Hydrogenase from Desulfovibrio Desulfuricans: A Q-Band 57Fe-ENDOR and HYSCORE Study.” *Journal of the American Chemical Society* 129 (37): 11447–58.

Silakov, Alexey, Brian Wenk, Eduard Reijerse, Simon P. J. Albracht, and Wolfgang Lubitz. 2009a. “Spin Distribution of the H-Cluster in the Hox–CO State of the [FeFe] Hydrogenase from Desulfovibrio Desulfuricans: HYSCORE and ENDOR Study of 14N and 13C Nuclear Interactions.” *JBIC Journal of Biological Inorganic Chemistry*. https://doi.org/10.1007/s00775-008-0449-5.
